# Early antimicrobial regimen shapes gut microbiota and health trajectories in pigs: a longitudinal study from weaning to finishing

**DOI:** 10.1186/s42523-025-00477-x

**Published:** 2025-10-24

**Authors:** F. Correa, D. Luise, G. Palladino, J. Estellé, S. Turroni, D. Scicchitano, G. Babbi, S. Rampelli, M. Candela, P. L. Martelli, C. Stefanelli, E. Perez-Calvo, Paolo Trevisi

**Affiliations:** 1 Department of Agricultural and Food Sciences, University of Bologna, 40127 Bologna, Italy; 2 Department of Pharmacy and Biotechnology, University of Bologna, 40126 Bologna, Italy; 3 Université Paris-Saclay, INRAE, AgroParisTech, GABI, Jouy-en-Josas 78350 France; 4 Department for Life Quality Studies, University of Bologna, 47921 Rimini, Italy; 5 Dsm-firmenich Animal Nutrition and Health, Kaiseraugst, 4303 Switzerland

**Keywords:** Microbial diversity, Oxidative stress, Metagenomics, Polyamines, AMR

## Abstract

This study investigated the longitudinal evolution of the gut microbiota, immune parameters, and antimicrobial resistance genes (ARGs) in pigs from pre-weaning to slaughter under two contrasting rearing conditions differing in antimicrobial use. At day (d) twenty-one postnatal, 96 piglets from 22 litters were assigned to SPC1 (Swine Production Chain 1) or SPC2 (Swine Production Chain 2) and maintained on the same stage specific diets until slaughter. Faecal and blood samples were collected at d21 (T1, pre-weaning), d42 (T2), d80 (T3), d98 (T4), and d278 (T5). Analyses included 16 S rRNA gene and shotgun faecal microbiome profiling (200 samples; 22 SPC1 and 18 SPC2 pigs), faecal polyamines, blood cell counts, immunoglobulins, oxidative stress markers, and ARGs profiling from metagenome-assembled genomes. From T1 to T2, all SPC1 pigs received group-level antimicrobials in water, whereas only seven SPC2 pigs were treated individually. At T2, SPC2 showed lower mean corpuscular haemoglobin concentration and monocytes but higher red blood cells, haemoglobin, and haematocrit (*P* < 0.05). At T3, SPC2 had higher red and white blood cells, basophils, and neutrophils but lower haemoglobin, haematocrit, and monocytes (*P* < 0.05). Gut microbial alpha diversity was significantly influenced by the Time × SPC interaction (*P* < 0.001). Key species differentiating SPC1 and SPC2 included *Megasphaera elsdenii*, *Lactobacillus johnsonii*, and *L. reuteri* (SPC2) versus *Prevotellaceae NK3B31* and *Rikenellaceae RC9* (SPC1) (LDA > 4, *P* < 0.01). Pathways for drug and xenobiotic metabolism were enriched in SPC1 at T3 (log2FC = 1.5, P adj. = 0.04). Dynamic Time Warping clustering revealed distinct microbial profiles in pigs with similar lymphocyte or neutrophil trajectories (R² = 0.002, *P* = 0.04; R² = 0.002, *P* < 0.01). ARG analysis revealed contrasting resistome patterns. In SPC1, metaphylactic treatment with lincomycin and spectinomycin led to transient enrichment of aminoglycoside resistance genes at T2 (log2FC = 2.1–2.4, P adj. < 0.05) and additional glycopeptide and multidrug genes at T3 (log2FC = 1.8–2.6, P adj. < 0.05), with no enrichments by T5. In SPC2, despite limited antimicrobial use, early and persistent enrichment was observed for MLS, tetracycline, aminoglycoside, beta-lactam, and multidrug resistance genes (log2FC = 1.7–3.2, P adj. < 0.05), many associated with mobile genetic elements. In conclusion, rearing practices, particularly antimicrobial protocols, shaped gut microbiota composition, immune responses, and the resistome from suckling to finishing.

**Supplementary Information:**

The online version contains supplementary material available at 10.1186/s42523-025-00477-x.

## Introduction

Pigs are a major source of meat worldwide [1], efficiently converting feed into animal protein and contributing substantially to the global food supply [2]. However, the pig production system also plays a central role in the One Health framework, particularly due to its implications for antibiotic use. The overuse of antibiotics in livestock contribute to the emergence of antibiotic-resistant bacteria, posing serious risks to both human and animal health [3]. To reduce reliance on antibiotics, the industry must implement effective alternative strategies. One promising approach is to improve pig robustness, defined as the animal’s ability to resist and adapt to challenges such as disease and environmental stressors with minimal medical intervention [4]. Enhancing robustness supports sustainable pig production and helps reduce the need for antibiotic treatments, ultimately benefiting both animal and human health.

In this context, it is continuously rising the awareness of the central role that the gastrointestinal microbiome can play for the host robustness. Since the initial postulation of Dubos et al. 1965 [5] who suggested that the intestinal microbiota and the host can interact and coevolve, several relevant scientific outcomes confirming this have been published. In young animals, including pigs, the gut microbiota is essential for the development of the host gut immune system [6, 7] and in later life, it play a significant role in the modulation of the host metabolism, for instance providing a number of important metabolites including vitamins [8], proving a re-cycling of bile salts [9] and contributing in the use of indigestible polysaccharides [10].

Moreover, emerging evidence highlights a complex interplay between the gut microbiota, behavioural responses, and inflammatory as well as oxidative stress [11]. The gut-brain axis—a bidirectional communication system linking the central nervous system and the gut microbiome—appears to influence both stress-related behaviours and physiological responses. Microbial-derived metabolites are capable of modulating neuroendocrine pathways and inflammatory processes, thereby impacting oxidative balance and behaviour [12].

The porcine gastrointestinal tract is an open and dynamic eco-system which is known to continually adapt to the activity of the host to the different environment and stimuli that it has during life. Indeed, several studies showed the dynamic evolution of the intestinal microbiota with the maturation and age of swine [13–15]. For instance, at birth microbial communities have a low diversity and richness that tend to increase during suckling. However, a decline in microbial diversity is observed immediately after weaning [16–18]. Subsequently, diversity increase during the growing stage but reduce significantly in finishing pigs (147-day-old pigs) compared to other growth stages [15]. Furthermore, several studies have identified a core microbiota and specific enterotypes associated with the age, maturations as well as growth, efficiency and immune parameters of pigs during the different stages of life [15, 17, 19, 20]. These results highlight the relevance of co-occurrence patterns of microorganisms and the key roles that microbial relationships play in the community assembly and stability driving various effects on the host health. However, it is also well recognised that external factors, including the diets and the environmental conditions, can modulate the microbial ecosystem. In addition, as reported by Trevisi et al. 2021 [21] each productive stage has its own influencing factors on pig gut microbiota. Indeed, it needs to be considered that the modern swine-rearing system had been adapted over time to be more efficient and standardized in order to optimize the resources, improve the piglet feed conversion efficiency and become more secure which is a prerequisite for livestock and meat production. As a consequence, the heavy pig rearing system is structured in three different production phases site 1 from birth to weaning, site 2 from weaning to 30 kg of weight and site 3 from 30 to 160 kg; these different stages can consequently be the source of direct or indirect stimuli to the host and in turn on the microbiota influencing their co-evolution and shifting.

The different management and environmental conditions—such as hygiene conditions, housing enrichment, and early antibiotic treatment— can affect pig health and productivity especially in the most critical phase of maturation including early life and weaning [22–24]. Saladrigas-García et al. 2022 showed that the farm environment and rearing systems significantly impact the gut microbiota development of young piglets with differences that were more noticeable after weaning than during lactation. Additionally, maternal influences on the initial colonization of the gastrointestinal tract greatly contribute to the evolution of the gut microbiota, with effects that can persist throughout the pig’s life [25]. However, studies specifically designed to estimate the effects of the environment and/or antibiotic use, while removing the maternal effect, and to link these modifications in gut microbiota to host health parameters including immune function and oxidative stress are lacking. Moreover, the available literature is mainly based on amplicon sequencing [23, 26], while data on shot-gun microbiome, that allow to have a comprehensive picture of the microbiome’s structure and functional potential, are still missing.

The aim of the study is to evaluate the evolution of the swine microbiome from the pre-weaning phase to slaughter under two different rearing conditions, which mainly differ for antibiotic use, to identify microorganisms or microbial patterns associated with health and physiological parameters throughout the swine production chain.

## Results

During the post-weaning phase, antimicrobial use differed markedly between the two production chains: SPC1 received a seven-day group treatment with lincomycin–spectinomycin, whereas in SPC2 antimicrobials were administered only to individually diagnosed pigs, involving marbofloxacin or amoxicillin–clavulanic acid.

### Diarrhoea, mortality, body lesions and growth performance

**Fig. 1:**
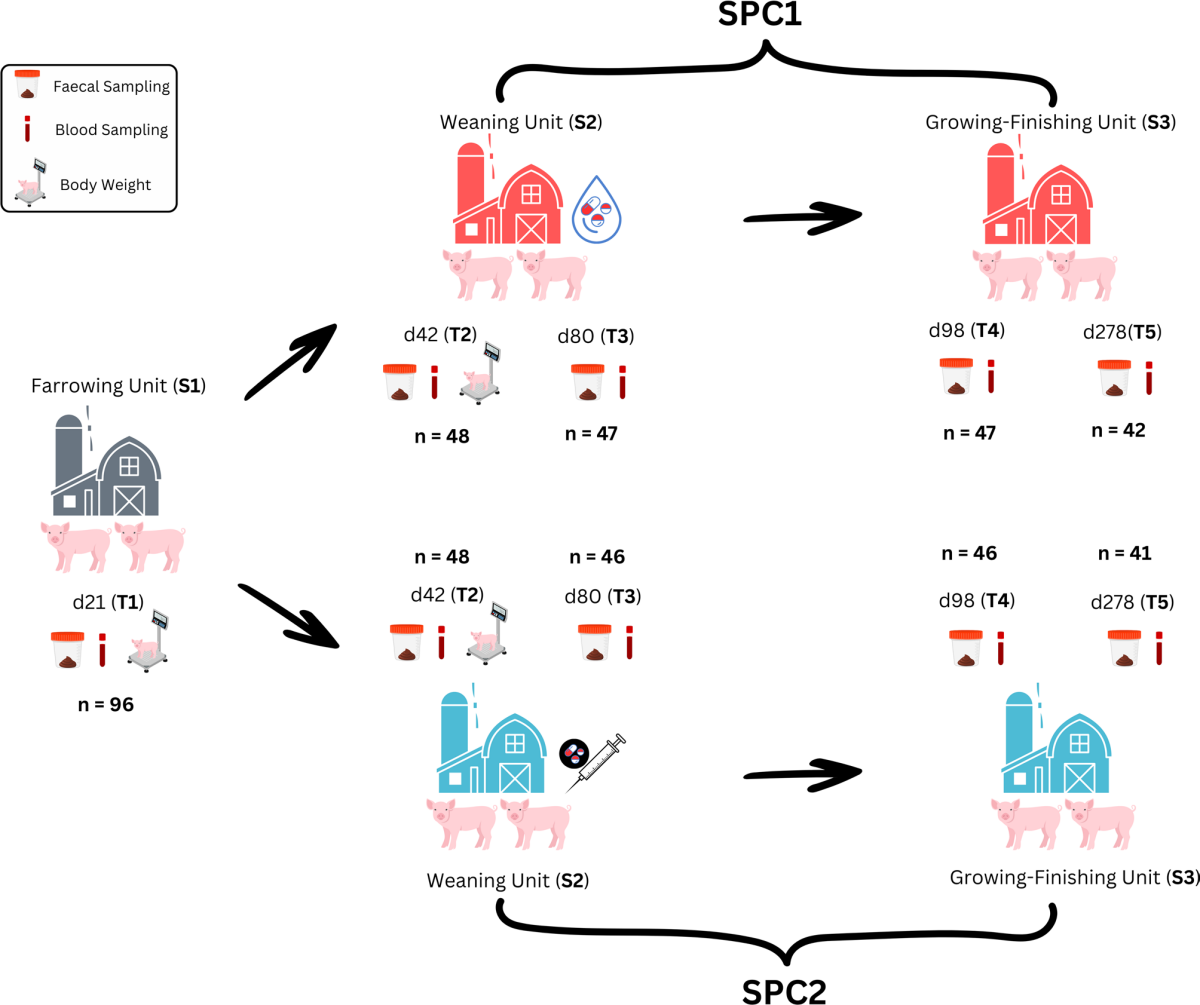
Diagram of the longitudinal study design comparing two swine production chains (SPC1 and SPC2)

A total of six pigs in SPC1 and seven pigs in SPC2 died during the trial. Specifically, in SPC1, one pig died between T2 and T3, and five died between T4 and T5. In SPC2, two pigs died between T2 and T3, and five died between T4 and T5. The overall study design and a summary of sampling performed is presented in Fig. 1. Data on diarrhoea occurrences are reported in Supplementary Fig. 1A. Pigs in SPC2 had a higher occurrence of diarrhoea at T2 compared to pigs of SPC1 (*P* < 0.01), no differences were observed in the other timepoints. Data on average daily gain (ADG) between T2 and day 80 (T3) are reported in Supplementary Fig. 1B; pigs in the SPC1 had a higher ADG compared to pigs in the SPC2 (*P* < 0.001).

Percentage of lesion scores (0, 1, 2) for six body parts (ear, neck, middle, hind quarters, tail and skin) of pigs at different timepoints (T2, T3, day 98 (T4), day 278 (T5)) between the two SSPCs are showed in Table 1. At T2, pigs in SPC1 had a higher incidence of lesion in ears (*P* = 0.03) and tails (*P* < 0.05), and a tendency for a higher lesion on the neck (*P* = 0.06), compared to SPC2. At T3, pigs in SPC1 had a higher incidence of lesions in ears (*P* = 0.02) and hind quarters (*P* = 0.04). At T4, pigs in SPC1 had a higher incidence of lesion in the tail (*P* = 0.01). At T5, pigs in SPC2 had a higher incidence of lesion in the tail (*P* = 0.01).

**Table 1:** Effect of swine production chain (SWP) on lesion incidence in ears, Hind quarters, middle, neck, skin and tail at different time points (T2, T3, T4, and T5)

Timepoint^1^	Body Part	SPC1			SPC2			P-value
		Score 0 (%)	Score 1 (%)	Score 2 (%)	Score 0 (%)	Score 1 (%)	Score 2 (%)	
T2	ears	75.00	22.73	2.27	50.00	36.36	13.64	**0.03**
	hind quarters	100.00	0.00	0.00	95.45	2.27	2.27	0.36
	middle	86.36	13.64	0.00	88.64	9.09	2.27	0.49
	neck	72.73	25.00	2.27	52.27	34.09	13.64	**0.06**
	skin	100.00	0.00	0.00	97.73	0.00	2.27	1.00
	tail	90.91	9.09	0.00	70.45	27.27	2.27	**< 0.05**
T3	ears	68.09	12.77	19.15	88.89	8.89	2.22	**0.02**
	hind quarters	85.11	14.89	0.00	93.33	2.22	4.44	**0.04**
	middle	65.96	27.66	6.38	73.33	17.78	8.89	0.51
	neck	65.96	23.40	10.64	66.67	22.22	11.11	0.99
	skin	100.00	0.00	0.00	100.00	0.00	0.00	0.83
	tail	82.98	14.89	2.13	71.11	22.22	6.67	0.34
T4	ears	84.00	10.00	6.00	90.91	9.09	0.00	0.25
	hind quarters	94.00	6.00	0.00	95.45	4.55	0.00	1.00
	middle	90.00	8.00	2.00	95.45	4.55	0.00	0.50
	neck	92.00	6.00	2.00	84.09	15.91	0.00	0.20
	skin	98.00	0.00	2.00	100.00	0.00	0.00	1.00
	tail	72.00	26.00	2.00	95.45	4.55	0.00	**0.01**
T5	ears	97.56	0.00	2.44	95.24	0.00	4.76	1.00
	hind quarters	95.12	4.88	0.00	90.48	9.52	0.00	1.00
	middle	90.24	7.32	2.44	80.95	19.05	0.00	0.31
	neck	78.05	17.07	4.88	90.48	4.76	4.76	0.39
	skin	100.00	0.00	0.00	100.00	0.00	0.00	1.00
	tail	100.00	0.00	0.00	76.19	23.81	0.00	0.01

^**1**^ T1 = 21 days of life; T2 = 42 days of life; T3 = 80 days of life, T4 = 98 days of life, T5 = 278 days of life

^a, b^ Values within a row with different superscripts differ significantly at *P *< 0.05. ^A, B^ Values within a row with different superscripts differ significantly at *P *< 0.01

### Blood parameters

Table 2 shows the effect of the time and SPCs on the blood cell counts. The interaction between SPCs and time significantly affected all the parameters (*P* < 0.05). Considering the comparison between SPC2 and SPC1 within each timepoint, no differences were observed at T1; at T2, the pigs in the SPC2 showed a lower level of mean corpuscular haemoglobin concentration (MCHC) and monocytes counts (*P* < 0.05) and a higher value of red blood cells (RBC), haemoglobin (HGB), haematocrit (HCT) (*P* < 0.05); at T3, the pigs in the SPC2 had a higher value of RBC, MCHC, white blood cell (WBC), basophils counts, neutrophils counts (*P* < 0.05) and a lower value of HGB, HCT, mean corpuscular haemoglobin (MCH), and monocytes counts (*P* < 0.05); at T4, the pigs in the SPC2 had a higher value of RBC, basophils counts, eosinophils counts (*P* < 0.05) and a lower value of MCH, MCHC, WBC, lymphocytes counts, monocytes counts (*P* < 0.05).

**Table 2:** Effect of the swine production chain, time and their interaction on the haematological parameters of pigs from weaning to fattening period. Values are reported as estimated marginal means ± standard error mean

Item^1^	Time^2^															*P* - value		
	T1^3^			T2			T3			T4			T5					
	SPC1	SPC2	SEM	SPC1	SPC2	SEM	SPC1	SPC2	SEM	SPC1	SPC2	SEM	SPC1	SPC2	SEM	SPC	Time	SPC x Time
RBC, M/µL	5.7	5.7	0.1	6.3B	6.9 A	0.1	6.9B	7.2 A	0.1	7.3B	7.7 A	0.1	7.3	7.3	0.2	0.64	**< 0.001**	**< 0.01**
HGB, g/d	11.9	11.8	0.2	10.4B	11.2 A	0.2	11.0 A	10.6B	0.2	11.6	11.4	0.1	13.9	13.9	0.3	0.55	**< 0.001**	**< 0.001**
HCT, %	43.4	43.0	0.7	37B	42.1 A	0.7	40.6 A	37.7B	0.7	42.6	43.2	0.7	39.6	38.8	1.1	0.54	**< 0.001**	**< 0.001**
MCH, pg	20.8	20.9	0.3	16.6	16.4	0.3	16.0 A	14.8B	0.2	15.9 A	14.9B	0.3	19.1	19.0	0.4	0.84	**< 0.001**	**< 0.01**
MCHC, g/dL	27.4	27.4	0.2	28 A	26.6B	0.2	27.2B	28.1 A	0.2	27.2 A	26.4B	0.2	35.1a	35.8b	0.4	0.93	**< 0.001**	**< 0.001**
MCV	76.0	76.1	0.6	59.1B	61.6 A	0.6	59.0 A	52.9B	0.6	58.3a	56.4b	0.6	54.5	53.0	1.2	0.83	**< 0.001**	**< 0.01**
WBC, K/µL	11.4	11.6	1.0	22.5	23.1	1.0	18.3 A	20.3B	1.0	22.2 A	19.9B	1.0	17.7	16.1	1.6	0.89	**< 0.001**	**< 0.05**
BASO, K/µL	0.15	0.16	0.03	0.24	0.20	0.03	0.16B	0.26 A	0.03	0.19B	0.39 A	0.03	0.29a	0.20b	0.1	0.8	**< 0.001**	**< 0.001**
EOSI, K/µL	0.07	0.07	0.06	0.30	0.23	0.06	0.33	0.37	0.06	0.33 A	0.64B	0.06	0.33	0.64	0.1	0.99	**< 0.001**	**< 0.001**
NEUTRO, K/µL	5.0	5.0	0.7	11.4	11.4	0.7	7.5B	9.9 A	0.6	11.4	10.6	0.6	6.0	6.0	1.0	0.95	**< 0.001**	**< 0.05**
LYMPHO, K/µL	6.0	6.2	0.6	10.1	10.8	0.5	9.9	9.4	0.5	9.8 A	7.9B	0.5	10.2	8.9	0.2	0.75	**< 0.001**	**< 0.05**
MONO, K/µL	0.18	0.21	0.03	0.48 A	0.39B	0.03	0.44 A	0.36B	0.03	0.51 A	0.32B	0.03	0.31	0.21	0.05	0.35	**< 0.001**	**< 0.001**
PLT, K/µL	498.0	442.0	25.1	548.0	575.0	24.3	517 A	502B	24.6	395 A	257B	25.4	288.0	265.0	47.4	0.08	**< 0.001**	**< 0.001**

^1^ RBC, red blood cells, HGB, haemoglobin, HCT, haematocrit, MCH, mean corpuscular haemoglobin, MCHC, mean corpuscular haemoglobin concentration, WBC, white blood cells, BASO, basophil, EOSI, eosinophil, NEUTRO, neutrophil, LYMPHO, lymphocytes, MONO, monocytes. ^1^Item: d-ROMs = Reactive Oxygen Metabolites; CARR U = Carratelli Units; BAP = Biological Antioxidant Potential; HP = Haptoglobin; CER = Ceruloplasmin; SAA = serum amyloid A; AOPP = Advanced Oxidative Protein Products; Ig = immunoglobulin. ^2^ T1 = 21 days of life; T2 = 42 days of life; T3 = 80 days of life, T4 = 98 days of life, T5 = 278 days of life ^3^ Contrast were performed within each Timepoint. ^a, b^ Values within a row with different superscripts differ significantly at *P *< 0.05. ^A, B^ Values within a row with different superscripts differ significantly at *P *< 0.01

Table 3 shows the effect of the time and SPCs on oxidative stress parameters and immunoglobulins concentration of pigs during the study. The interaction between SPCs and time significantly affected all the parameters (*P* < 0.05), except the concentration of serum amyloid A (SAA) and immunoglobulin M (IgM) which were affected only by the time (*P* < 0.0001); both SAA and IgM concentration increased with time. At T1, the piglets in the SPC2 had a higher concentration of biological antioxidant potential (BAP), haptoglobin (HP), advance oxidation protein products (AOPP) (*P* < 0.05) therefore, for these parameters, the concentration at T1 was used as a covariate for the analysis at T2. At T2, the piglets in the SPC2 had a lower concentration of reactive oxygen metabolites (ROMs), and a higher concentration of BAP and HP (*P* < 0.05). At T3, the piglets in the SPC2 had a higher concentration of BAP (*P* < 0.05) while they had a lower concentration of ceruloplasmin (CER) (*P* < 0.05). At T4, the pigs in the SPC2 had a lower concentration of ROMs (*P* < 0.05); At T5, the pigs in the SPC2 had a lower concentration of BAP, HP and IgM (*P* < 0.05).

**Table 3:** Effect of the swine production chain, time and their interaction on oxidative parameters Immunoglobulins concentration and faecal polyamines of from weaning to fattening period. Values are reported as estimated marginal means ± standard error mean

Item^1^	Time^2^															*P*-value		
	T1^3^			T2			T3			T4			T5					
	SPC1^3^	SPC2	SEM	SPC1	SPC2	SEM	SPC1	SPC2	SEM	SPC1	SPC2	SEM	SPC1	SPC2	SEM	SPC	Time	SPC x Time
d-ROMs, CARRU	273.0	276.0	4.6	261.0 A	246.0B	4.5	253.0	254.0	4.5	276.0 A	250.0B	4.3	266.0	271.0	10.4	0.62	**< 0.01**	**< 0.01**
BAP, µmol/L	1558B	1777 A	34	1825B	2110 A	34	1929B	2092 A	34	2512	2459	33	2067 A	1888B	78	**< 0.001**	**< 0.001**	**< 0.001**
HP, µg/ml	35.6 A	28.2B	0.9	38.3B	42.9 A	0.9	46.7	48.0	0.9	47.2	45.6	0.9	49.6 A	39.0B	2.1	**< 0.001**	**< 0.001**	**< 0.001**
CER, ng/ml	14.4	18.2	5.6	69.7	67.2	5.5	135.3B	97.2 A	5.4	116.3	120.0	5.3	170.7	160.8	12.8	0.61	**< 0.001**	**< 0.001**
SAA, ng/ml	21.1	31.5	12.1	28.7	36.6	11.9	79.8	100.5	11.8	119.1	123.9	11.4	389.7a	337.7b	28.1	0.53	**< 0.001**	0.34
AOPP, nmol/mg	7.2B	8.7 A	0.3	2.7	2.6	0.3	2.0	1.5	0.3	1.4	2.1	0.3	1.6	1.3	0.7	**< 0.001**	**< 0.001**	**< 0.05**
IgA, ng/mL	0.18	0.20	0.00	0.84	0.71	0.00	1.35b	1.57a	0.10	1.86	1.69	0.10	1.35	1.00	0.3	0.88	**< 0.001**	0.06
IgM, ng/mL	0.71	0.50	0.30	2.16a	1.65b	0.30	2.77	2.82	0.30	3.07	2.55	0.30	6.42 A	5.16B	0.7	0.48	**< 0.001**	0.40
IgG, ng/mL	14.2b	19.8a	2.4	9.4	9.3	2.4	10.2	15.4	2.3	20.0	16.2	2.3	19.4	11.4	6.0	0.09	**< 0.01**	0.08
Polyamine/mL																		
Putrescine	543.1	536.0	145.0	590.0	377.5	152.0	269.5	415.0	147.0	250.4	205.9	145.0	45.2	101.9	273.0	0.97	**< 0.05**	0.80
Cadaverine	2876 A	1464B	478	978	1722	500	636	1523	483	375	393	478	87	129	904	**< 0.05**	**< 0.001**	0.11
Spermidine	306.0	354.0	27.2	595 A	442B	28.5	639 A	562B	27.5	419.0	387.0	27.2	313.0	403.0	51.5	0.20	**< 0.001**	**< 0.01**
Spermine	30.6	25.4	4.3	75.8 A	47.4B	4.5	93.5 A	62.9B	4.3	31.4	24.5	4.3	14.8	22.4	8.1	0.37	**< 0.001**	**< 0.001**

^1^Item: d-ROMs = Reactive Oxygen Metabolites; CARR U = Carratelli Units; BAP = Biological Antioxidant Potential; HP = Haptoglobin; CER = Ceruloplasmin; SAA = serum amyloid A; AOPP = Advanced Oxidative Protein Products; Ig = immunoglobulin. ^2^T1= 21 days of life; T2 = 42 days of life; T3 = 80 days of life, T4 = 98 days of life, T5 = 278 days of life. ^3^ Contrast were performed within each Timepoint.^a, b^ Values within a row with different superscripts differ significantly at *P *< 0.05. ^A, B^ Values within a row with different superscripts differ significantly at *P *< 0.01

### Faecal polyamine

The effect of the time and SPCs on faecal polyamine concentration is reported in Table 3. The interaction between SPCs and time significantly affected the spermidine and spermine concentrations (*P* < 0.001), while time significantly influenced all the polyamines (*P* < 0.05). The SPCs significantly influenced the cadaverine concentration (*P* = 0.03) and it was mainly visible at T1 when the piglets in the SPC1 had a higher cadaverine concentration than piglets in SPC2. Furthermore, both at T2 and T3, the SPC1 site had higher concentration of spermine and spermidine (*P* < 0.05). No differences were observed between the two SPCs at T4 and T5.

### Microbiota profile

**Fig. 2:**
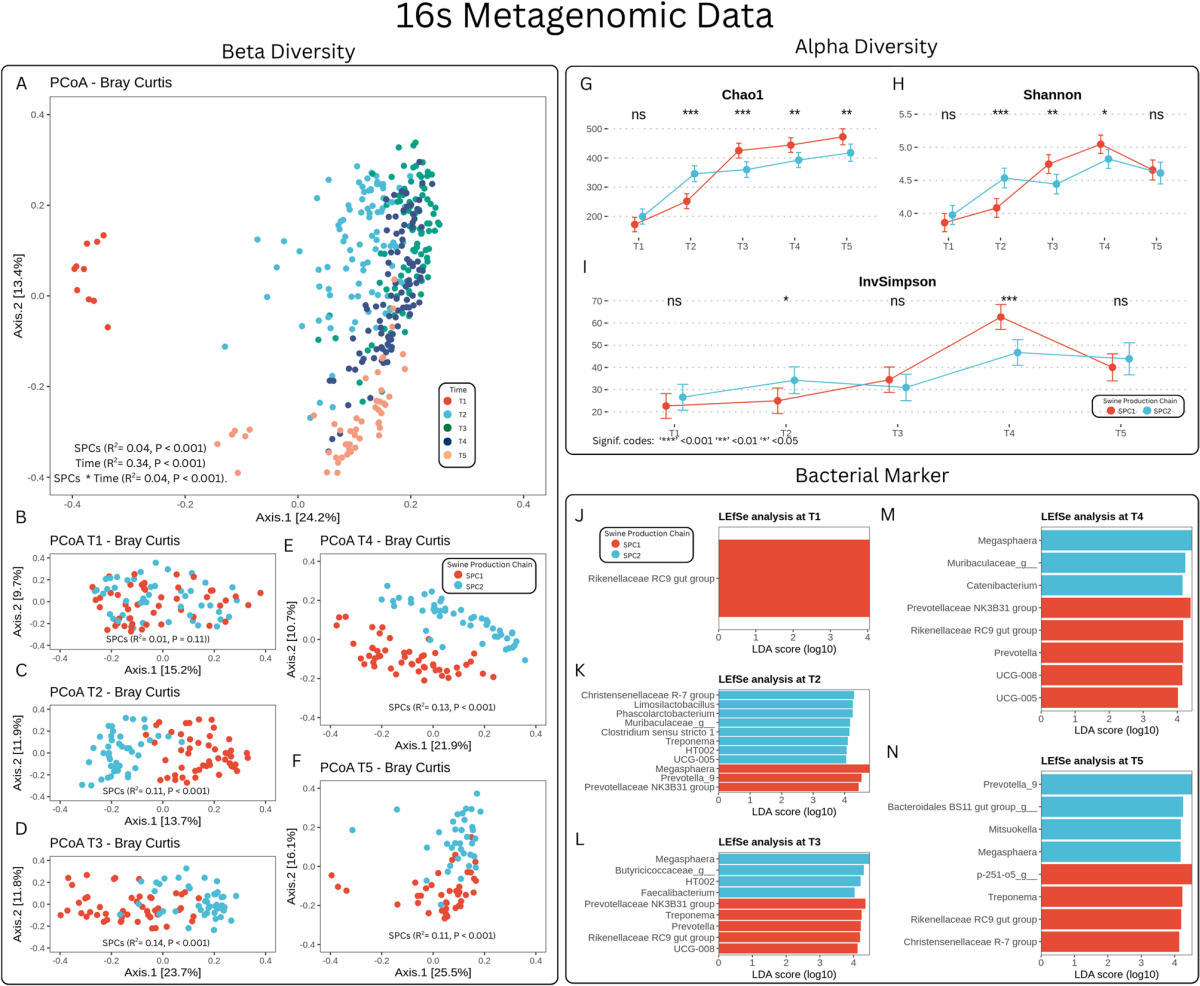
Microbiota structure based on 16S rRNA amplicon sequencing across time and production. (**A**) Principal coordinate analysis (PCoA) based on Bray–Curtis distances showing clustering of samples by timepoint. (**B**–**F**) PCoA plots by timepoint comparing SPC1 and SPC2. (**G**–**I**) Alpha diversity indices (Chao1, Shannon, InvSimpson) across time and production chains. (**J**–**N**) LEfSe plots of genera discriminating between SPC1 and SPC2 at T1–T5

For 16 S sequencing, a total of 12,655,201 reads were attributed to a total of 16,947 amplicon sequence variants (ASVs) distributed among samples. The taxonomic assignment allowed obtaining 31 phyla, 246 families and 678 genera. The alpha diversity indices were significantly affected by time (Chao1, *P* < 0.001; Shannon, *P* < 0.001 and InvSimpson, *P* < 0.001) and by the interaction between SPCs and time (Chao1, *P* < 0.001; Shannon, *P* < 0.001 and InvSimpson, *P* < 0.001) (Fig. 2G, H and I). At T1, no differences were observed between SPCs for all the alpha diversity indices considered. At T2, pigs of SPC1 had a lower richness (Chao1, *P* < 0.001) and alpha diversity (Shannon, *P* < 0.001, InvSimpson, *P* = 0.02) compared to the SPC2. At T3, SPC1 had a higher richness (Chao1, *P* < 0.001) and Shannon index (*P* < 0.01) compared to the SPC2. At T4, the SPC1 had a higher Chao1 index (*P* = 0.01), Shannon (*P* < 0.01) and InvSimpson (*P* = 0.03) indices compared to the SPC2. At T5, no differences were recorded for the diversity indices.

For the beta diversity, the Principal Coordinate Analysis (PCoA), Fig. 2A shows that samples tend to separate based on their maturity since they clusterize according to the time of sampling. Beta diversity was affected by SPCs (R^2^ = 0.04, *P* < 0.001), time (R^2^ = 0.34, *P* < 0.001) and their interaction (R^2^ = 0.04, *P* < 0.001). In addition, considering the effect of SPCs (SPC1 and SPC2) within each timepoint (Fig. 2B, C, D, E and F), the PCoA plot and the Adonis analysis showed that the beta diversity was not affected by the SPCs at T1, when piglets were reared in the same farm, but it was significantly affected by the SPCs at T2 (*P* = 0.001, R^2^ = 0.11), T3 (*P* = 0.001, R^2^ = 0.14), T4 (*P* = 0.001, R^2^ = 0.13) and T5 (*P* = 0.001, R^2^ = 0.11).

Microbial markers characterizing each SPCs are reported in Fig. 2. At T1, Rikenellaceae RC9 gut group (LDA = 4.01, P adj. < 0.01) was significantly more abundant in pigs from SPC1 compared to SPC2 (Fig. 2J). At T2, *Megasphaera* (LDA = 4.74, P adj. < 0.001), *Prevotella*_9 (LDA = 4.53, P adj. < 0.01), and Prevotellaceae NK3B31 group (LDA = 4.46, P adj. < 0.001) had a significantly higher abundance in SPC1 pigs. Conversely, several genera including Christensenellaceae R-7 group (LDA = 4.28, P adj. < 0.001), *Limosilactobacillus* (LDA = 4.27, P adj. < 0.001), *Phascolarctobacterium* (LDA = 4.23, P adj. < 0.001), Muribaculaceae (LDA = 4.15, P adj. < 0.001), *Clostridium sensu stricto* 1 (LDA = 4.13, P adj. < 0.001), HT002 (LDA = 4.07, P adj. = 0.006), *Treponema* (LDA = 4.06, P adj. < 0.001), and UCG-005 (LDA = 4.05, P adj. < 0.001), had a higher abundance in SPC2 pigs (Fig. 2K).

At T3, pigs in SPC1 had a higher abundance of Prevotellaceae NK3B31 group (LDA = 4.31, P adj. < 0.001), *Treponema* (LDA = 4.28, P adj. < 0.001), *Prevotella* (LDA = 4.25 P adj. < 0.001), Rikenellaceae RC9 gut group (LDA = 4.16, P adj. < 0.001) and of UCG-008 (LDA = 4.09, P adj. < 0.001). In contrast, pigs in SPC2 had higher levels of *Megasphaera* (LDA = 4.51, P adj. = 0.0095), Butyricicoccaceae (LDA = 4.26, P adj. < 0.001), uncultured genera of HT002 (LDA = 4.23, P adj. < 0.001) and *Faecalibacterium* (LDA = 4.05, P adj. < 0.001) (Fig. 2L).

At T4, Prevotellaceae NK3B31 group (LDA = 4.47, P adj. < 0.001), *Prevotella* (LDA = 4.21, P adj. < 0.001), Rikenellaceae RC9 gut group (LDA = 4.21, P adj. < 0.001), UCG-008 (LDA = 4.20, P adj. < 0.001), and UCG-005 (LDA = 4.13, P adj. < 0.001) were significantly more abundant in pigs from SPC1. *Megasphaera* (LDA = 4.51, P adj. = 0.0001), *Catenibacterium* LDA = 4.15, P adj. < 0.001), and an uncultured genera from Muribaculaceae (LDA = 4.18, P adj. < 0.001) were significantly more abundant in pigs from SPC2 (Fig. 2M).

At T5, p-251-o5 (LDA = 4.57, P adj. < 0.001), Rikenellaceae RC9 gut group (LDA = 4.25, P adj. = 0.0006), *Treponema* (LDA = 4.23, P adj. = 0.0099), and Christensenellaceae R-7 group (LDA = 4.04, P adj. = 0.0045) were significantly more abundant in pigs from SPC1 group. In contrast, *Prevotella*_9 (LDA = 4.49, P adj. < 0.001), *Megasphaera* (LDA = 4.18, P adj. = 0.0015), *Bacteroidales* BS11 gut group (LDA = 4.17, P adj. < 0.001), and *Mitsuokella* (LDA = 4.12, P adj. < 0.001) were significantly more abundant in pigs from SPC2 (Fig. 2N).

### Metagenomic taxonomic profile

By Illumina shotgun sequencing of the faecal microbial DNA from the selected pigs, a total of 1.476 billion paired-end reads were generated, with an average of 7.418 million (± 4.081 million SD) reads per subject. The core microbiota analysis revealed marked temporal differences in microbial composition (Supplementary Fig. 2 and Supplementary Table 1). Core members were defined as those taxa that reached a relative abundance of at least 1% in individual samples and were present in at least 50% of samples at each time point. At T1, 12 core taxa were identified, including *Methanobrevibacter smithii*, *Butyricimonas virosa*, *Alistipes shahii*, *Parabacteroides merdae*, *Lactobacillus crispatus*, *Lactobacillus reuteri*, *Lactobacillus vaginali*s, *Clostridium clostridioforme*, *Anaeromassilibacillus sp. An172*, *Ruminococcaceae bacterium D5*, *Phascolarctobacterium succinatutens*, and *Escherichia coli*. At T2, four core taxa were identified: *Prevotella copri*, *Lactobacillus amylovorus*, *Lactobacillus reuteri*, and *Megasphaera elsdenii*. At T3, eight core taxa were identified, including *Prevotella copri*, *Lactobacillus amylovorus*, *Lactobacillus johnsonii*, *Lactobacillus reuteri*, *Butyricicoccus porcorum*, *Coprococcus catus*, *Phascolarctobacterium succinatutens*, and *Megasphaera elsdenii*. At T4, nine core taxa were identfied: *Collinsella aerofaciens*, *Prevotella copri*, *Prevotella sp. CAG 520*, *Lactobacillus amylovorus*, *Lactobacillus johnsonii*, *Lactobacillus reuteri*, *Butyricicoccus porcorum*, *Phascolarctobacterium succinatutens*, and *Megasphaera elsdenii*. At T5, eight core taxa were: *Methanobrevibacter smithii*, *Prevotella copri*, *Prevotella sp. P5 92*, *Lactobacillus amylovorus*, *Lactobacillus reuteri*, *Coprococcus catus*, *Turicibacter sanguinis*, and *Treponema porcinum*. The intersection of the core taxa across timepoints T1 through T5 revealed that only one taxon was consistently present: *Lactobacillus_reuteri*. Between timepoints T2 to T5, three taxa were consistently detected: *Prevotella copri*, *Lactobacillus amylovorus*, and *Lactobacillus reuteri*.

**Fig. 3:**
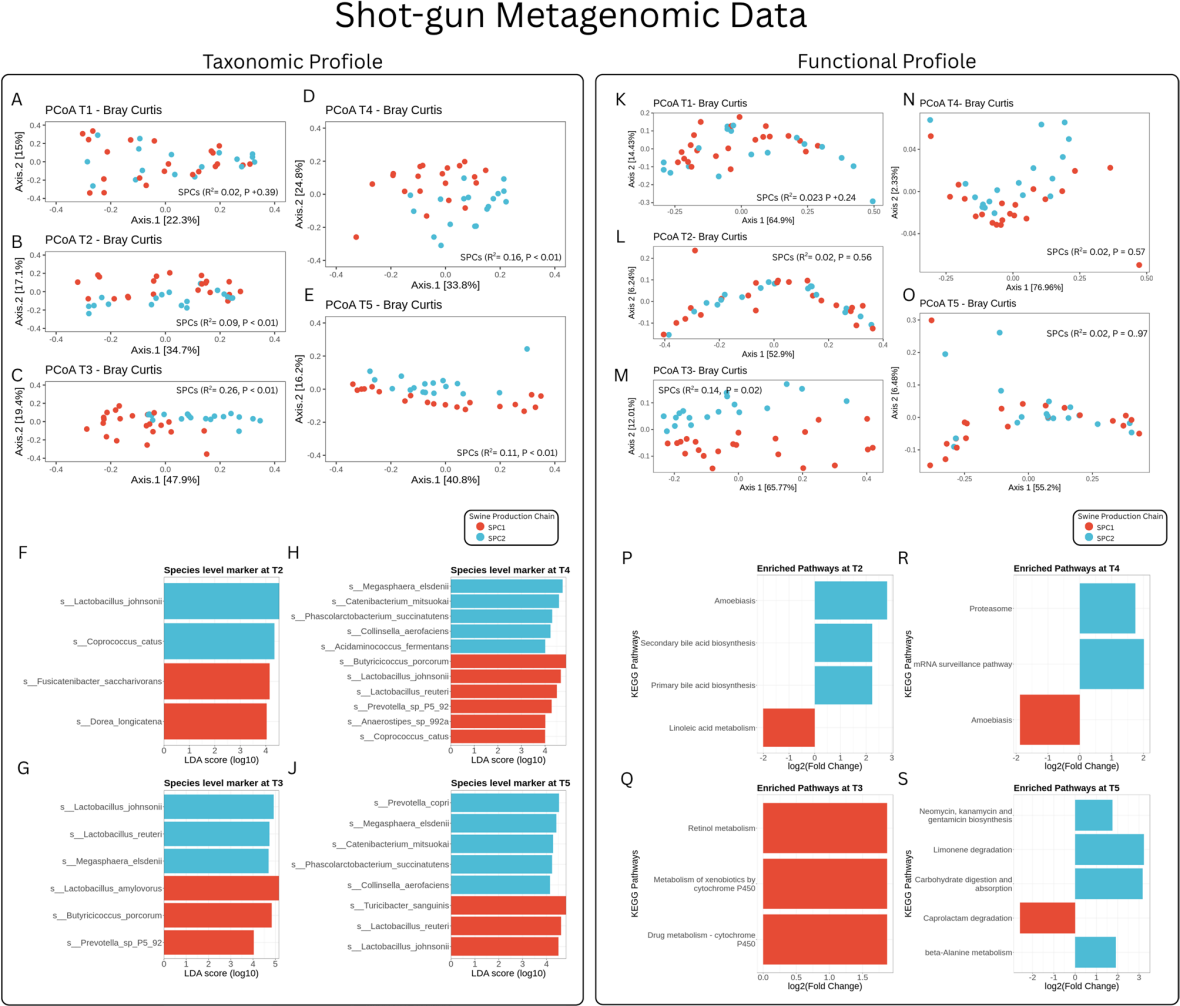
Metagenomic taxonomic and functional profiles of pigs across production chains. (**A**–**E**) PCoA plots of species-level taxonomic profiles from shotgun metagenomics. (**F**–**J**) LEfSe plots of bacterial species significantly associated with SPC1 or SPC2 at T2–T5. (**K**–**O**) PCoA of KO-level microbial functional profiles by production chain and timepoint. (**P**–**S**) Differentially abundant KEGG pathways identified using DESeq2 for each timepoint

Moreover, in order to prove that the selected samples were representatives of the entire cohort they were drawn from, the PcoA on Bray-Curtis distance matrix at Species level was reported (Fig. 3A, B, C, D and E). The Adonis analysis showed a similar pattern to the 16 S Amplicon analysis with the beta diversity that was not affected by the SPCs at T1 (*P* = 0.39, R^2^ = 0.02), when piglets were reared in the same farm, but it was significantly affected by the SPCs at T2 (*P* < 0.01, R^2^ = 0.09), T3 (*P* < 0.01, R^2^ = 0.26), T4 (*P* < 0.01, R^2^ = 0.16) and T5 (*P* < 0.01, R^2^ = 0.11). LEfSe analysis was used to identify bacterial species associate to the different production chains over time (Fig. 3F, G, H, and J). At T1, no differences were observed between SPC1 and SPC2, while at T2 *Fusicatenibacterm saccharivorans* (LDA = 4.14, P adj. < 0.001) and *Dorea longicatena* (LDA = 4.00, P adj. < 0.001) were significantly more abundant in pigs from SPC1 pigs, whereas pigs from SPC2 had a higher abundance of *Lactobacillus johnsonii* (LDA = 4.52, P adj. < 0.001) and *Coprococcus catus* (LDA = 4.35, P adj. < 0.001). At T3, *Lactobacillus amylovorus* (LDA = 5.19, P adj. < 0.001) and *Butyricicoccus porcorum* (LDA = 4.78, P adj. < 0.001) were more abundant in pigs from SPC1 group, while the SPC2 pigs had a higher abundance of *Lactobacillus johnsonii* (LDA = 4.93, P adj. < 0.001), *Lactobacillus reuteri* (LDA = 4.72, P adj. < 0.001), and *Megasphaera elsdenii* (LDA = 4.68, P adj. < 0.01). At T4, *Butyricicoccus porcorum* (LDA = 4.84, P adj. < 0.01), *Lactobacillus johnsonii* (LDA = 4.64, P adj. < 0.001), *Lactobacillus reuteri* (LDA = 4.55, P adj. < 0.01), Prevotella sp_P5_92 (LDA = 4.22, P adj. < 0.01), *Coprococcus catus* (LDA = 4.06, P adj. < 0.001), and *Anaerostipes* sp_992a (LDA = 4.02, P adj. < 0.001) were significantly more abundant in the SPC1 pigs, while *Megasphaera elsdenii* (LDA = 4.74, P adj. < 0.001), *Catenibacterium mitsuokai* (LDA = 4.65, P adj. < 0.001), *Phascolarctobacterium succinatutens* (LDA = 4.32, P adj. < 0.001), *Collinsella aerofaciens* (LDA = 4.29, P adj. < 0.001), and *Acidaminococcus fermentans* (LDA = 4.04, P adj. < 0.001) were significantly more abundant in the SPC2 pigs. At T5, *Turicibacter sanguinis* (LDA = 4.90, P adj. = 0.0071), *Lactobacillus reuteri* (LDA = 4.56, P adj. = 0.0056), *Desulfovibrio piger* (LDA = 4.00, P adj. < 0.001) and *Lactobacillus johnsonii* (LDA = 4.41, P adj. < 0.001) were significantly more abundant in the SPC1 pigs while in the SPC2 group *Megasphaera elsdenii* (LDA = 4.42, P adj. < 0.001), *Prevotella copri* (LDA = 4.39, P adj. = 0.0023), *Catenibacterium mitsuokai* (LDA = 4.26, P adj. < 0.001), *Colinsella aerofaciens* (LDA = 4.00, P adj. < 0.001) and *Phascolarctobacterium succinatutens* (LDA = 4.25, P adj. < 0.001) were significantly more abundant in the SPC2 pigs.

### Metagenomic functional profile

The functional characterization of the faecal microbiota was performed using HUMAnN2 to quantify the abundance of microbial functions based on different levels of KEGG (Kyoto Encyclopedia of Genes and Genomes) classification. PCoA based on Bray-Curtis distances of functional gene profiles at KEGG orthology (KO) level showed a SPCs related differences (Fig. 3K, L, M, N and O). In particular, Adonis test showed that the KO profiles were not different between pigs in the SPC1 and SPC2 at T1 (*P* = 0.24, R^2^ = 0.03), T2 (*P* = 0.56, R^2^ = 0.02), T4 (*P* = 0.57, R^2^ = 0.02), T5 (*P* = 0.97, R^2^ = 0.02), but it was significantly affected by the SPCs at T3 (*P* = 0.02, R^2^ = 0.14). DESEQ2 was used to identify pathways significantly associated to each SPCs in the different timepoints (Fig. 3P, Q, R and S).

For pigs from SPC1, at T2, their functional profile was characterized by pathways related to “Linoleic acid metabolism” (Log2FC = -2.01, *P* < 0.05). At T3, pigs from SPC1 showed functional enrichment in pathways related to “Biosynthesis of unsaturated fatty acids” (Log2FC = 1.87, *P* < 0.05), “Metabolism of xenobiotics by cytochrome P450” (Log2FC = 1.87, *P* < 0.05), and “Drug metabolism – cytochrome P450” (Log2FC = 1.87, *P* < 0.05), no specific pathways were associated with SPC2 at T3. At T4, the pigs from SPC1 showed enrichment in pathways related to “Amoebiasis” (Log2FC= -1.89, *P* < 0.05), whereas “mRNA surveillance pathways” (Log2FC = 2.01, *P* < 0.05) and the “Proteasome” (Log2FC = 1.75, *P* < 0.01) were more abundant in SPC2. By T5, the functional profile of pigs in SPC1 was dominated by pathways related to “Caprolactam degradation” (Log2FC= -2.59, *P* < 0.01), whereas in SPC2 pathways related to “beta-Alanine metabolism” (Log2FC = 1.91, *P* < 0.001), “Neomycin, kanamycin and gentamicin biosynthesis” (Log2FC = 1.75, *P* < 0.01), “Limonene degradation” (Log2FC = 3.23, *P* < 0.001), and “Carbohydrate digestion and absorption” (Log2FC = 3.17, *P* < 0.001) were more abundant.

### AMR genes profiles

**Fig. 4:**
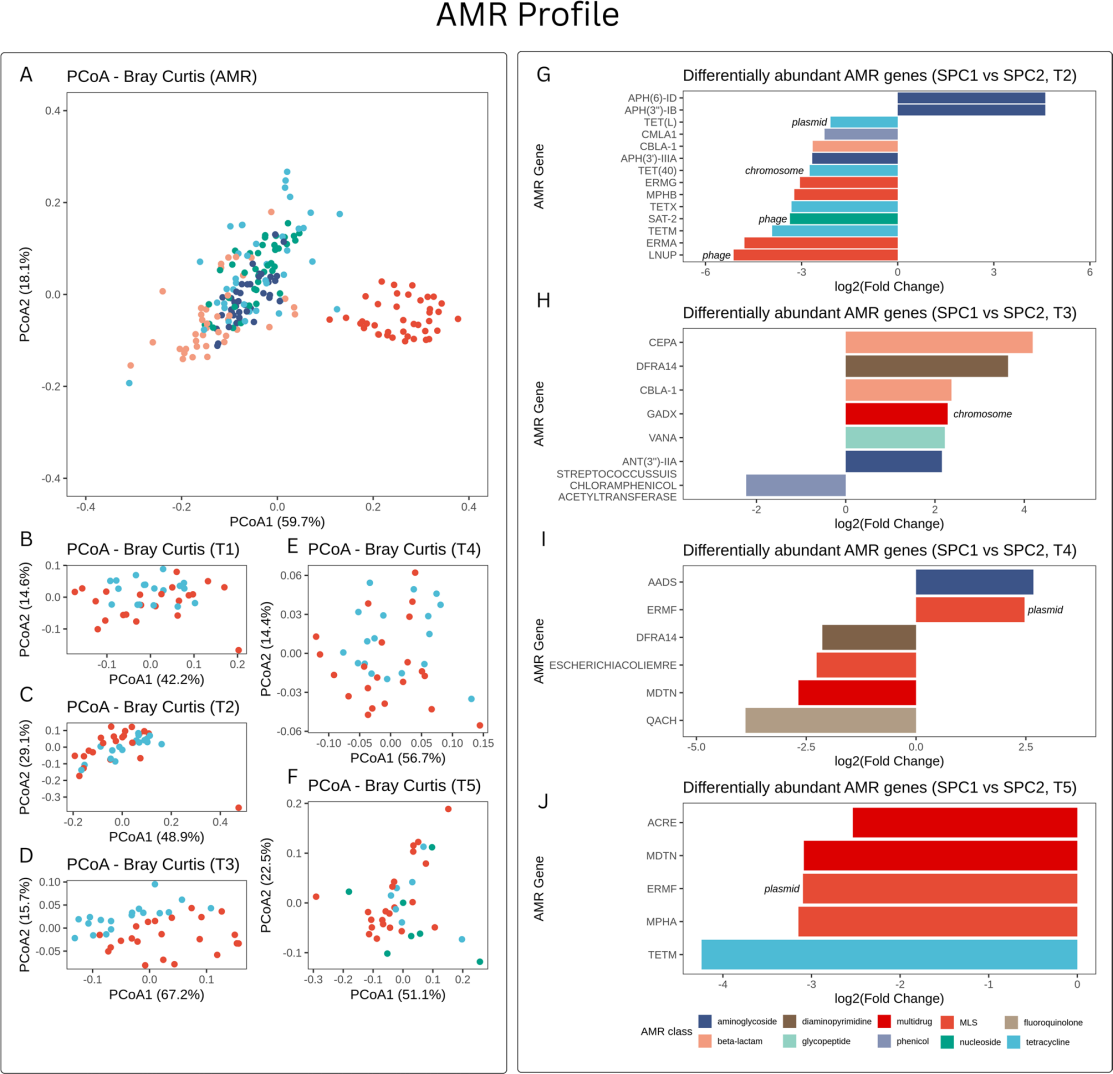
Temporal dynamics of antimicrobial resistance gene profiles between production chains. (**A**) PCoA of AMR gene profiles across all timepoints. (**B**–**F**) PCoA by timepoint comparing SPC1 and SPC2. (**G**–**J**) Differential abundance of AMR genes at T2–T5 identified using DESeq2

Among the 35 metagenome-assembled genomes (MAGs) identified a total of 168 ARGs were identified. The PCoA plots based on Bray-Curtis distances are reported in Fig. 4. The PERMANOVA model highlighted a strong effect of time on AMR gene composition (*P* < 0.001, R² = 0.55), and a significant interaction between SPC and time (*P* < 0.001, R² = 0.03), suggesting that the influence of the SPC on the resistome varied across timepoints. The main effect of the SPC do not show any significant effect (*P* = 0.10, R² = 0.005) (Fig. 4A). Further pairwise comparisons at each timepoint revealed no significant differences between SPC1 and SPC2 at T1 (*P* = 0.19, R² = 0.04) (Fig. 4B), whereas trends were observed at T2 (*P* = 0.08, R² = 0.05) (Fig. 4C), and significant differences emerged from T3 onward: T3 (*P* < 0.01, R² = 0.20) (Fig. 4D), T4 (*P* < 0.05, R² = 0.07) (Fig. 4E), and T5 (*P* < 0.05, R² = 0.09) (Fig. 4F).

Difference in AMR gene abundance is reported in Fig. 4. At T1 no differences in AMR gene abundance were found. At T2 (Fig. 4G), pigs from SPC2 exhibited significantly higher abundance of LNUP (Log2FC = -5.12, *P* < 0.001), classified under Macrolide–Lincosamide–Streptogramin (MLS) resistance and detected on phage elements; *ERMA* (Log2FC = -4.78, *P* < 0.001), also in the MLS class; and *TETM* (Log2FC = -3.92, *P* < 0.001), a tetracycline resistance gene. Other genes enriched in SPC2 included SAT-2 (Log2FC = -3.36, *P* = 0.001), a nucleoside resistance gene associated with phage; *TETX* (Log2FC = -3.31, *P* < 0.001; tetracycline class); *MPHB* (log2FC = -3.23, *P* = 0.002; MLS); and ERMG (Log2FC = -3.05, *P* < 0.001; MLS). The tetracycline resistance gene *TET(40)* (Log2FC = -2.75, *P* < 0.001) was enriched in SPC2 and associated with chromosomal elements. Additional genes with higher abundance in SPC2 included *APH(3’)-IIIA* (Log2FC = -2.67, *P* < 0.001; aminoglycoside class), *CBLA-1* (Log2FC = -2.65, *P* = 0.003; beta-lactam), CMLA1 (Log2FC = -2.28, *P* < 0.001; phenicol), and *TET(L)* (Log2FC = -2.10, *P* < 0.001; tetracycline), the latter located on plasmids. Conversely, pigs from SPC1 showed higher levels of *APH(3’’)-IB* and *APH(6)-ID* (both log2FC = 4.61, *P* < 0.001), two aminoglycoside resistance genes.

At T3 (Fig. 4H), SPC2 pigs exhibited greater abundance of *STREPTOCOCCUS SUIS CHLORAMPHENICOLACETYLTRANSFERASE* (log2FC = -2.23, *P* < 0.001; phenicol class). While pigs from SPC1 had a higher abundance of *ANT(3’’)-IIA* (log2FC = 2.15, *P* < 0.001; aminoglycoside), *VANA* (log2FC = 2.22, *P* = 0.008; glycopeptide), and *GADX* (log2FC = 2.28, *P* < 0.001; multidrug class), the latter mapped to chromosomal regions. Additional enriched genes in SPC2 included *CBLA-1* (log2FC = 2.37, *P* = 0.016; beta-lactam) and *DFRA14* (log2FC = 3.64, *P* < 0.001; diaminopyrimidine).

At T4 (Fig. 4I), SPC1 pigs had higher levels of ERMF (log2FC = 2.47, *P* < 0.001; MLS), which was located on plasmids, and *AADS* (log2FC = 2.67, *P* = 0.003; aminoglycoside class). On the other hand, SPC2 pigs exhibited higher abundance of QACH (log2FC = -3.88, *P* < 0.001; fluoroquinolone class), *MDTN* (log2FC = -2.68, *P* < 0.001; multidrug), *ESCHERICHIACOLIEMRE* (log2FC = -2.26, *P* < 0.001; MLS), and DFRA14 (log2FC = -2.13, *P* < 0.001; diaminopyrimidine).

At T5 (Fig. 4J), the resistome in SPC2 pigs was characterized by increased abundance of TETM (log2FC = -4.24, *P* < 0.001; tetracycline), *MPHA* (log2FC = -3.15, *P* < 0.001; MLS), *ERMF* (log2FC = -3.10, *P* < 0.001; MLS; plasmid-borne), *MDTN* (log2FC = -3.09, *P* < 0.001; multidrug), and *ACRE* (log2FC = -2.53, *P* < 0.001; multidrug). No ARGs were significantly enriched in SPC1 at this timepoint.

### Association between blood parameters and microbiota

**Fig. 5:**
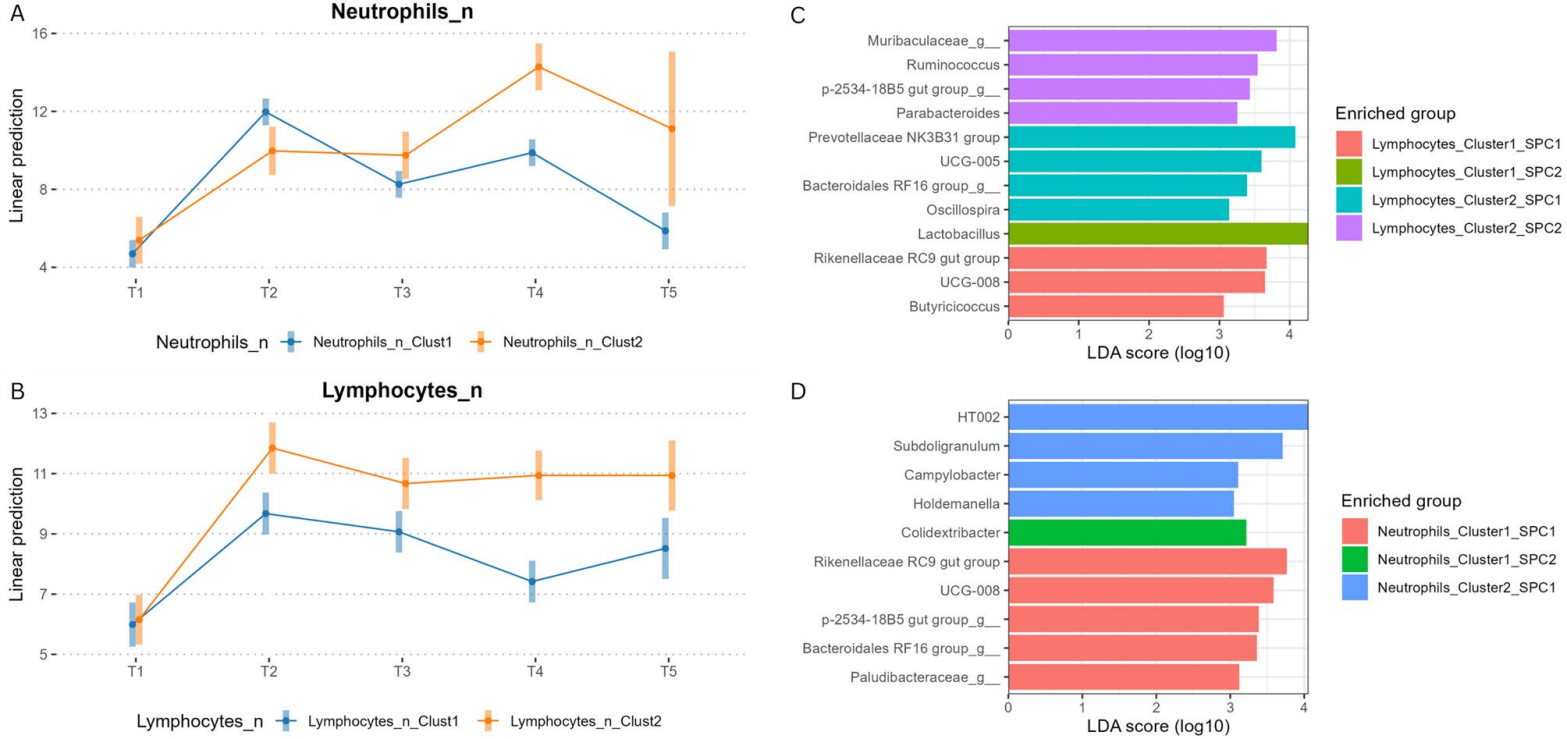
Association between blood immune parameters and faecal microbiota. (**A**–**B**) Dynamic time warping (DTW)-based clustering of neutrophil and lymphocyte trajectories across the study. (**C**–**D**) LEfSe analysis showing bacterial genera associated with lymphocyte clusters in SPC1 and SPC2. (**E**–**F**) LEfSe plots of bacterial taxa associated with neutrophil clusters in both production chains

To evaluate the relationship between blood cell counts and microbiota composition, hierarchical clustering was applied to Dynamic Time Warping (DTW) distances to identify temporal patterns in blood parameters, both individually for each blood parameters and globally using average DTW distances. Among the blood clusters derived using the DTW distance, only those associated with lymphocytes (R² = 0.002, *P* = 0.04) and neutrophils (R² = 0.002, *P* < 0.01) exhibited a significant interaction effect with SPCs on the 16 S microbial profile beta-diversity analyses. The trajectories of lymphocyte and neutrophil counts for each cluster are showed in Fig. 5A and B. Specifically, two clusters were identified for neutrophils and they had similar neutrophil counts level at T1, while the cluster 1 had a higher neutrophil counts at T2 and from T3 to T5 had a lower neutrophil count level than cluster 2. The two lymphocytes clusters identified displayed the same lymphocyte levels at T1; however, pigs from cluster 1 showed consistently lower lymphocyte levels from T2 to T5 compared to those from cluster 2. Furthermore, the pairwise Adonis tests revealed that pigs belonging to cluster 1 of neutrophils had a significantly different microbial profile on SPC1 compared to SPC2 (R² = 0.002, *P* < 0.05). Conversely, the microbial profile of pigs from cluster 2 did not differ significantly. Additionally, pigs belonging to cluster 1 of lymphocytes in SPC1 exhibited a different microbial profile compared to pigs from the same cluster in SPC2 (R² = 0.002, P adj. < 0.05), and similarly, pig from cluster 2 of lymphocytes in SPC1 showed a different microbial profile compared to pigs of the same cluster in the SPC2 (R² = 0.002, P adj. < 0.05). However, no interaction effects were observed between SPCs and either the lymphocytes or neutrophils clusters on the alpha diversity indices.

Results of the LEfSe analysis (Fig. 5C and D) evidenced that for lymphocytes in SPC1, four bacterial taxa were significantly enriched in cluster 1: Rikenellaceae RC9 gut group (LDA score = 3.67, P adj. < 0.001), UCG-008 (LDA score = 3.65, P adj. < 0.001), and *Butyricicoccus* (LDA score = 3.06, P adj. < 0.05). In contrast, *Lactobacillus* was enriched in cluster 1 of lymphocytes in SPC2 (LDA score = 4.27, P adj. < 0.05). For the cluster 2 of lymphocytes, in the SPC1 four bacterial taxa were enriched: Prevotellaceae NK3B31 group (LDA score = 4.08, P adj. < 0.001), Bacteroidales RF16 group (LDA score = 3.39, P adj. < 0.001), *Oscillospira* (LDA score = 3.14, P adj. < 0.001), and *UCG-005* (LDA score = 3.60, P adj. < 0.001), while the cluster 2 of lymphocytes in SPC2 the following four taxa were significantly enriched: Muribaculaceae (LDA score = 3.81, P adj. < 0.001), p-2534-18B5 gut group (LDA score = 3.43, P adj. < 0.05), *Parabacteroides* (LDA score = 3.25, P adj. < 0.001), and *Ruminococcus* (LDA score = 3.54, P adj. < 0.05).

For neutrophils in SPC1, five bacterial taxa were significantly enriched in cluster 1: Rikenellaceae RC9 gut group (LDA score = 3.77, P adj. < 0.05), UCG-008 (LDA score = 3.58, P adj. < 0.05), p-2534-18B5 gut group (LDA score = 3.39, P adj. < 0.05), Bacteroidales RF16 group (LDA score = 3.36, P adj. < 0.05), and Paludibacteraceae (LDA score = 3.12, P adj. < 0.05). No significant taxa were detected for cluster 1 in the SPC2. Regarding the cluster 2 of neutrophils, in the SPC1 *Colidextribacter* was enriched (LDA score = 3.22, P adj. < 0.05), while for the SPC2, four bacterial taxa were significantly enriched: *HT002* (LDA score = 4.06, P adj. < 0.01), *Subdoligranulum* (LDA score = 3.71, P adj. < 0.001), *Campylobacter* (LDA score = 3.11, P adj. < 0.01), and *Holdemanella* (LDA score = 3.05, P adj. < 0.001).

## Discussion

This study provides new insights into how commercial rearing conditions, and particularly the antibiotic use, can influence the development of gut microbiota, immune function, and oxidative stress from birth to slaughter in pigs. Unlike previous investigations limited to weaning [17, 24] or focused solely on later growth phases [26], our longitudinal design allowed to monitor the same animals across key developmental transitions under controlled litter origin and diet, isolating the effects of rearing conditions.

During the suckling period, when piglets were reared together and shared a common environment, no clinical health issues were reported. This was confirmed by haematological parameters, which showed no significant differences between animals, except for initial variations in BAP, haptoglobin, AOPP, and IgG levels, which were accounted for in subsequent analyses. Examining the effect of time on haematological parameters during the suckling and weaning phases, the number of white blood cells, lymphocytes, neutrophils, and basophils increased steadily from the suckling period to weaning. After this period, these values stabilized, indicating the maturation of the adaptive immune system. This development was initially suppressed during the suckling phase due to the presence of maternal IgG, which was reflected in elevated IgG levels in the piglets’ blood [27]. Interestingly, suckling piglets exhibited higher levels of ROMs, indicating increased oxidative stress. This observation was further supported by elevated levels of AOPP, which are proteins modified through oxidation primarily by reactive oxygen species. The oxidative stress is likely linked to the transition from intrauterine to extrauterine life, during which neonates experience an oxidative burst and require time to establish antioxidant balance [28]. This process may be exacerbated in fast-growing pig lines, which have a higher metabolic rate compared to slower-growing genetic lines [29]. Regarding erythrocyte parameters, during the suckling phase, piglets exhibited a low RBC count alongside elevated mean corpuscular volume (MCV) and MCH, this can be a sign of the presence of cells of erythroid origin still under maturation. This condition, commonly observed in suckling piglets [30], is attributed to the limited iron content of sow’s milk. Despite the administration of iron dextran injections during the early days of life, piglets still experienced anaemia, indicating that early supplementation may not have been sufficient to fully prevent this condition [31].

Considering the effect of rearing conditions on the faecal microbiome, results showed no significant difference in the microbiome structure during the lactation period when pigs were reared together. However, after this period, the environment significantly influenced microbiome diversity and composition, this effect was observed for both 16 S and shotgun metagenomics data. Examining the evolution of alpha diversity metrics over time, the faecal microbiome gradually acquired greater diversity in terms of taxa composition, but this as observed in our study, was not linked to an increase in the functional diversity. This phenomenon is referred as functional redundancy, which allows the microbiome to cope with and adapt to environmental changes and refers to the presence of multiple microbial species capable of performing similar ecological roles, ensuring the stability of essential functions despite shifts in microbial composition [25].

Despite changes in bacterial taxa composition, the core microbiota analysis identified core bacterial members that were defined as having a minimum relative abundance of at least 1% in individual samples and were present in at least 50% of samples at each timepoint. At T1, 39% of the core taxa were present only at this time point. These included *Butyricimonas virosa*, *Alistipes shahii*, *Parabacteroides merdae*, *Lactobacillus crispatus*, *Lactobacillus vaginalis*, *Clostridium clostridioforme*, *Anaeromassilibacillus sp. An172*, *Ruminococcaceae bacterium D5*, and *Escherichia coli*. This reflects how the pre-weaning gut microbiota of piglets is dominated by bacteria specialized in utilizing milk oligosaccharides [32]. Notably, *Lactobacillus vaginalis*, a bacterium typically isolated from the vaginal tract of humans and sows [33, 34], was the most common and abundant species during the suckling period. This suggests that the initial colonizers of the gastrointestinal tract from the birth canal play a substantial role in shaping the piglet microbiota during early life. Moreover, *Lactobacillus reuteri* was consistently present across all time points, this suggest how some taxa can persist as core members of the microbiota from sucking until slaughter [35]. Considering members of the core microbiota from the weaning phase, *Prevotella copri* and *Lactobacillus amylovorus* were the core taxa present in all pigs from T2 to T5, indicating a how core microbiota from weaning is dominated by fibre-degrading bacteria [17]. The presence of this taxa as the most abundant in pigs gut microbiota was already observed in previous studies [36, 37].

Following weaning, when piglets were moved to the different rearing environments and subjected to distinct antimicrobial protocols, the impact of these management differences on host physiology and microbiota composition became evident. Pigs in SPC1 received a metaphylactic antibiotic treatment via drinking water during the initial post-weaning period to control post-weaning diarrhoea (PWD), whereas in SPC2, the treatment was restricted to a few pigs with clinical symptoms. By day 10 post-weaning, pigs in SPC2 exhibited a higher incidence of diarrheal, more ears and tails lesions, and reduced growth compared to those in SPC1 suggesting that the metaphylactic treatment in SPC1 contributed to improved early growth by mitigating inflammatory and infectious stressors [38]. This early advantage in SPC1 was reflected in immune and oxidative stress markers. Ten days after weaning, pigs in this group showed elevated IgM levels and increased d-ROMs, accompanied by reduced antioxidant defences. These results indicate, on one hand, a successful activation of the adaptive immune system in response to early post-weaning sanitary challenges, and on the other hand, suggest that the associated inflammatory response may have contributed to an oxidative imbalance. This is consistent with the known interplay between oxidative stress and immune activation in pigs [39], where reactive oxygen metabolites often rise during the early phases of immune stimulation. By 7 weeks post-weaning, oxidative stress markers indicated that the antioxidant system in SPC1 pigs had not yet fully recovered. This was evidenced by significantly lower BAP levels and elevated ceruloplasmin levels, a copper-containing acute-phase protein synthesized in the liver. The increase in ceruloplasmin a protein whose ferroxidase activity plays a role in iron homeostasis and protection against free radicals—is often associated with prolonged oxidative stress. These findings suggest that while SPC1 pigs may have benefitted from early immune activation and pathogen control, this came with a physiological trade-off in redox balance that persisted well beyond the immediate post-weaning period.

In contrast, pigs in SPC2, who did not receive the metaphylactic antibiotic treatment, likely experienced enteric infections in the first ten days after weaning. This was corroborated by higher blood levels of HP, a marker of acute inflammation, and a higher haematocrit value, which can be linked to dehydration caused by PWD [40]. Additionally, in this period pigs in SPC2 exhibited a higher stress levels linked to aggressive behaviours, as evidenced by the increased incidence of lesions on their tails and ears. This behavioural pattern is consistent with previous findings that link health impairment and immune activation with increased aggressive behaviours in weaned pigs [41, 42]. Moreover, our previous study evidence how body lesions linked to aggressive behaviours negatively impacts the physiological homeostasis of weaned pigs by altering their oxidative status leading to immune function suppression and increased production of free radicals, resulting in an imbalance in blood oxidation and antioxidation [30]. Interestingly, despite greater early health challenges, SPC2 pigs displayed a stronger immune profile by seven weeks post-weaning, with higher white blood cell counts and IgA levels. This suggests that, while delayed, their immune response was eventually robust, potentially reflecting a more gradual adaptation to microbial exposure in the absence of broad-spectrum antimicrobial suppression.

Microbiota analysis revealed corresponding differences. At T2, alpha diversity was lower in SPC1, likely due to the selective pressure of the in-water antibiotic treatment [18, 42, 43]. Six weeks after weaning, the microbiome of the siblings in the two SPCs continued to differ in terms of alpha diversity. Although diversity recovered by six weeks post-weaning, increases were primarily observed in metrics sensitive to rare taxa (Chao1 and Shannon), but not in the InvSimpson index, which emphasizes dominant species. Moreover, this suggests that the gut microbial ecosystem recovered after the antibiotic treatment, but the ecosystem did not reach a stable composition. Such partial recovery is consistent with findings from both pig and murine models, where short-term lincomycin exposure—a lincosamide-class antibiotic used in SPC1—leads to marked shifts in microbiota composition followed by gradual re-equilibration over several weeks [44, 45].

Considering the taxa characterizing each SPC in the weaning phase, pigs in SPC1 were characterized by *Prevotella* or other Prevotellaceae subspecies. This genus is the most abundant in the post-weaning phase and is known to ferment complex carbohydrates deriving from the switch from a milk-based to a plant-based diet [16]. In contrast, pigs in SPC2 were characterized by HT002, a member of the Lactobacillaceae, which are mostly abundant in the suckling phase [16]. This suggests delayed microbial maturation in SPC2, possibly due to the inflammatory and nutritional consequences of PWD [43]. On the other hand, looking at the taxa identified with 16s amplicon data which characterized each SPC over time, it can be observed that bacteria from Rikennellaceae RC 9 gut group and Prevotellaceae were consistently more abundant in pigs of the SPC1, while *Megapshera* was consistently more abundant in pigs of the SPC2. Both Rikennellaceae RC 9 gut group and Prevotellaceae are part of the Bacteroidales and are important acetate producers forming part of the microbiota of pigs [44]. Species-level metagenomic analysis allowed to identify that *Megasphera elsedenii* was more abundant in pigs from SPC2, which is a bacterium that is normally found in the gastrointestinal tracts of pigs. It plays a crucial role in fermenting lactate and producing short-chain fatty acids (SCFAs), predominantly valerate [45]. But, when supplemented with lactic acid producing bacteria, it can increase the butyrate production [46]. In the present study, we observed that the SPC2 had a higher abundance of *Megasphera elsedenii*,* L. jhonsoniii* and *L. reuteri*; these results can support the hypothesis that *Megasphera elsedenii* takes advantage of the presence of Lactobacilli that produce lactate using it as a substrate for his metabolism.

In terms of functional potential, SPC2 microbiomes showed increased expression of pathways related to primary and secondary bile acid metabolism at day 10 post-weaning. This function, present in only a limited subset of gut anaerobes [49], plays a critical role in host-microbe signalling and gut homeostasis. In contrast, pigs in SPC1—who experienced PWD and received antibiotics—had reduced representation of these pathways, suggesting a disruption of key metabolic capacities. This may suggest that the metabolism of bile acids was probably reduced in piglets in SPC1 that experience PWD-related symptoms in this phase and received an in-water antimicrobial treatment.

Six weeks after the antibiotic treatment, pigs in SPC1 were characterized by microbiota that expressed more genes related to drug and xenobiotics metabolism. This functional enrichment suggests that the antimicrobial exposure in early post-weaning selected for microbial taxa capable of tolerating or metabolizing antimicrobial compounds, indicating a shift toward a community with increased resistance potential [46]. Together with the marked differences in terms of bacterial taxa and functional potential, also the microbial faecal samples of pigs from the SPC1 had a consistently lower spermine and spermidine in the faeces during the post-weaning period. Spermine and spermidine are metabolites that are produced in the gut mainly by gut bacteria via fermentation of dietary protein [47]. Since pigs in the SPC1 grew significantly more compared to pig of SPC2 in the ten days post-weaning, they probably had a higher feed intake suggesting a higher presence of substrate available for polyamine production by microbial fermentation.

Transitioning to the growing-finishing phase, pigs faced cumulative immunological, metabolic, and environmental stressors that stemmed from earlier differences in rearing conditions. While haematological values measured at T4 remained within physiological reference ranges [48], significant differences emerged in oxidative stress and immune markers. In particular, pigs from SPC1 showed elevated levels of white blood cells, lymphocytes, and d-ROMs. Notably, an increase in d-ROM, white blood cells and lymphocytes serum concentration was detected at T4 in pigs raised in SPC1. This rise in oxidative stress and immune response markers could be associated with the increase in tail lesions observed in this group, which may indicate an increase in aggressive behaviours. The T4 sampling point, which occurred closer to the transition from the weaning to the growing-finishing unit, likely reflected the stress of the transport and later on of the adaptation to a new environment, that might have played a role in the manifestation of tail lesions [49]. Our previous study [26], has linked impaired antioxidant systems to aggression in pigs, suggesting that oxidative stress might exacerbate or result from such behaviours. Similarly, in a murine model exposure to a social stressor, like social disruption, a significantly change in the community structure of the intestinal microbiota with a loss in in microbiota alpha diversity that was also associated with increased inflammatory cytokines (IL-6 and MCP-1) [50]. These findings underscore the importance of the gut-brain-immune axis in stress-related health outcomes.

Considering the effect of the SPC on the potential metabolic activity in the growing-finishing period, the mRNA surveillance and the proteasome pathway were significantly activated in the microbiota of pigs reared in SPC2. The mRNA surveillance pathway ensures defective mRNA degradation in eukaryotes via mechanisms like nonsense-mediated decay. In bacteria, mRNA degradation involves small regulatory RNAs and protein factors. Similarly, while the proteasome pathway is prominent in eukaryotes, bacteria use alternative proteolytic systems for protein degradation. These pathways are critical for maintaining cellular function, particularly in processes like bacterial development, sporulation, and pathogenesis. Further analysis of the microorganisms involved in these two pathways identified *Methanobrevibacter smithii* as the dominant player in both mRNA surveillance and proteolytic processes. This archaeon is well-documented in the pig microbiome and has been positively correlated with increased body weight during the finishing stage [51]. Its involvement is linked to its ability toinfluence the production of SCFAs, and to promote fat deposition by enhancing energy harvest from the diet [52]. At the end of the finishing period, pigs fed the SPC2 diet exhibited a marked enrichment in the microbial pathways associated with carbohydrate digestion and absorption as well as aminoglycoside biosynthesis, particularly those for neomycin, kanamycin, and gentamicin. Taxonomic contribution analysis revealed that these pathways were predominantly driven by *Megasphaera elsdenii*, a taxon that was consistently abundant in the SPC2 group from weaning through finishing. Although the aminoglycoside biosynthesis pathway is classically linked to *Streptomyces* and related Actinobacteria in soil ecosystems, its enrichment in the pig gut microbiota likely reflects functional annotation overlap rather than true antibiotic production. In fact, the pathway enrichment observed here appears to be largely attributed to the presence of two KOs: K00844, coding for hexokinase (EC 2.7.1.1), and K12047, coding for maltase-glucoamylase (EC 3.2.1.20; 3.2.1.3). These enzymes are central to the hydrolysis and phosphorylation of carbohydrates, processes that support microbial energy harvest and/or are a key mechanism for this taxa to use simple carbohydrate present in the mucus layer and colonize gut mucosa, a process already observed in humans [53].

In contrast, the microbiota of pigs raised in SPC1 exhibited a notable association with genes related to the amebiasis pathway in KEGG during the growing-finishing period. While this pathway typically involves *Entamoeba histolytica* and its interactions with host cells, in our study, these genes were predominantly contributed by *Coprococcus catus*, which was found to be higher in these pigs during the growing finishing period. *C. catus* is a commensal bacterium in the pig gut and is a significant producer of butyrate, that plays a crucial role in maintaining gut health by supporting colonocyte function and exerting anti-inflammatory effects [54].

Looking at the abundance of AMR genes detected in the MAGs we can highlight how the different antimicrobial treatment implemented in the two production chains contributed to the resistome patterns observed. In SPC1, pigs received a metaphylactic group treatment during the post-weaning period with lincomycin and spectinomycin administered via drinking water. This approach was associated with an early enrichment in aminoglycoside resistance genes such as APH(3’’)-IB and APH(6)-ID at T2, which are likely linked to spectinomycin exposure. At T3, additional genes including ANT(3’’)-IIA (aminoglycoside), VANA (glycopeptide), and GADX (multidrug class) were enriched in SPC1 pigs, suggesting a broadening of resistance potential beyond the classes directly targeted by the administered antimicrobials. At T4, higher levels of ERMF (MLS class; plasmid-associated) and AADS (aminoglycoside) were detected, but by T5 no ARGs were significantly enriched in this group, indicating a possible attenuation of selective pressure or stabilization of the resistome.

In contrast, SPC2 followed a more restrictive antimicrobial protocol, with only seven pigs receiving treatment based on individual clinical signs. Nonetheless, the resistome in SPC2 pigs exhibited early and consistent enrichment of a broader array of ARGs. By T2, genes conferring resistance to MLS (*LNUP*, *ERMA*, *MPHB*, *ERMG*), tetracyclines (*TETM*, *TETX*, *TET(40)*), aminoglycosides (*APH(3’)-IIIA*), beta-lactams (*CBLA-1*), phenicols (*CMLA1*), and diaminopyrimidines (*DFRA14*) were already more abundant in SPC2 animals. Many of these genes were associated with mobile genetic elements such as phages and plasmids. This trend persisted through T3 and T4, during which additional enrichment in *QACH* (fluoroquinolone), *MDTN* and *ACRE* (multidrug resistance), and recurrent detection of *DFRA14* further highlighted the diverse and mobilizable nature of the SPC2 resistome. By T5, SPC2 pigs maintained higher levels of *TETM*, *MPHA*, *ERMF*, *MDTN*, and *ACRE*, underscoring a consolidated pattern of tetracycline, MLS, and multidrug resistance.

Overall, although pigs in SPC1 were exposed to a broader antimicrobial pressure through group-level metaphylactic treatment, their resistome remained more limited and was largely restricted to the period immediately following treatment. In contrast, the more selective and targeted use of antimicrobials in SPC2 was associated with a broader and more persistent array of resistance genes, many of which were linked to mobile genetic elements. These findings suggest that factors beyond direct antibiotic exposure may play a key role in shaping the resistome. Notably, as highlighted in a previous study conducted within the same experimental framework [55], which investigated the environmental dissemination of ARGs, the farm environment—including manure, litter, water, and surfaces—may act as a reservoir for antimicrobial resistance genes, contributing to their persistence and transmission.

Furthermore, as environment-host-microbiota are strongly interrelated and changes in the environment can directly and indirectly modify both immune status and microbiota, the present study further investigated the relationship between blood cell count parameters and faecal microbiota over time. The results showed that although there are clusters of pigs characterised by low or high levels of lymphocytes, lymphocyte levels can be associated with different faecal microbiota profiles. Notably, animals in lymphocyte cluster 2, which includes pigs with higher lymphocyte counts from weaning to the growing-finishing phase, were characterized by bacteria such as Prevotellaceae NK3B31 and UCG-05, uncultivated genera of the Ruminococcaceae family, in SPC1, while in SPC2 these pigs were characterized by a higher abundance of *Ruminococcus*. *Ruminococcus* has been associated with increased immune system activation in humans [56], and members of the Prevotellaceae family have been linked to enhanced immune responses and vaccination efficacy in pigs [57].

In contrast, pigs of the Neutrophil cluster 2, comprising animals with higher neutrophil counts during the growing-finishing phase, were characterized by the presence of opportunistic pathogens such as *Campylobacter* in SPC1. This bacterial genus includes species known to cause gastrointestinal inflammation and systemic infections, which may trigger neutrophil recruitment as part of the host’s innate immune response [58]. In SPC2, no taxa were significantly associated with this neutrophil cluster, suggesting potential differences in microbial–immune interactions between production chains.

Although our study design does not allow us to establish causality, these findings are consistent with studies showing mechanistic links between microbiota and immune regulation. For example, Belkaid and Hand (2014) [59] highlighted that microbial-associated molecular patterns —such as lipopolysaccharides flagellin, and peptidoglycans—can activate innate immune receptors like Toll-like receptors, promoting neutrophil recruitment and cytokine production. Similarly, Round and Mazmanian (2009) [60] demonstrated that bacterial molecules, such as *Bacteroides fragilis* polysaccharide A, promote regulatory T cell differentiation and modulate pro-inflammatory pathways, supporting our finding that distinct microbiotas might lead to same immune phenotype.

## Conclusions

In conclusion, this study provides valuable insights into how commercial rearing conditions, and particularly antibiotic use, shape the gut microbiota, immune function, and health of pigs across developmental stages. While early-life microbial composition remains relatively stable during suckling, significant structural and functional shifts occur post-weaning, influenced notably by environmental factors and antibiotic regimens. Importantly, our findings highlight the functional resilience of the gut microbiome—its ability to maintain or recover key metabolic capacities despite taxonomic variability. This resilience was evident in the microbiota’s capacity to regain diversity and restore core functions following antibiotic intervention, even when compositional differences linked to rearing practices persisted. These observations underscore the dynamic interplay between gut microbiota, immune development, and environmental inputs. They also support the need for refined antibiotic strategies and targeted rearing management. Further studies should investigate the complex interactions between host immunity, microbiota, and rearing environments, while leveraging precision farming (i.e., use of prebiotics or probiotics) to define personalized strategies tailored to specific rearing conditions to optimize animal behaviour and welfare.

## Methods

### Experimental design

The in vivo trial was conducted in northern Italy using two swine production chains (SPC1 and SPC2), each involving a distinct combination of commercial farms. The production system included one shared farrowing unit (S1), two weaning units (S2), and two growing–finishing units (S3). A schematic representation of the experimental design is provided in Fig. 1.

At 21 days of age (T1), a total of 96 piglets (Landrace × Large White; 5,841 ± 799 g body weight) were selected from 24 sows (four piglets per sow) within the same farrowing unit (S1). The sows had the following parity distribution: 6 of parity 2, 3 of parity 3, 6 of parity 4, 1 of parity 5, 2 of parity 6, 6 of parity 7, and 2 of parity 8. To control for the influence of the maternal environment on early microbiota development, piglet selection and allocation were balanced by litter of origin, with two piglets per litter assigned to each production chain (SPC1 and SPC2).

Piglets were individually weighed, and ear tagged. At weaning (28 days of age), piglets were balanced by body weight and litter of origin, then equally allocated into two weaning farms (S2). Each group was subsequently transferred to a corresponding growing–finishing unit (S3). These combinations defined two distinct production chains: SPC1 and SPC2. All pigs were slaughtered at the same abattoir at the end of the trial.

To minimize confounding effects on microbiota composition, pigs in both chains were fed the same diets throughout the trial. A three-phase diet was used during the weaning period (S2), and a four-phase diet during the growing–finishing period (S3), as detailed in Supplementary Tables 2 and 3.

In both SPC1 and SPC2 weaning unit, pigs were housed in a single pen per group on fully slatted concrete floors (SPC1) or partially slatted (SPC2). Environmental conditions were automatically controlled to maintain appropriate temperature, ventilation system was natural in SPC1 while it was natural/forced in SPC2 weaning units. Feed and water were provided ad libitum through automatic feeders and nipple drinkers. Environmental enrichment was minimal, consisting of iron chains combined with wooden blocks. Space allowance was approximately 0.35 m² per pig, in accordance with the requirements of European Directive 2008/120/EC. Both weaning farms implemented an all-in/all-out management system. At the end of each cycle, the barns were thoroughly cleaned and disinfected following a standardized protocol. After disinfection, the facilities were left empty for one week before the arrival of the next group of piglets.

During the post-weaning phase, antimicrobial protocols differed markedly between the two production chains. In SPC1, all pigs received a group treatment via drinking water consisting of lincomycin combined with spectinomycin (15 mg/kg body weight) for seven consecutive days. The choice of active substances was guided by clinical evaluation of PWD symptoms and aligned with national and EU guidelines on prudent antimicrobial use, with diagnostic testing performed when deemed appropriate. In SPC2, antimicrobials were administered only to pigs individually diagnosed with PWD, following prescription by the designated farm veterinarian. Two pigs received a single intramuscular injection of marbofloxacin (8 mg/kg body weight), and five pigs were treated for five consecutive days with amoxicillin combined with clavulanic acid (7 mg/kg body weight, once daily). All antimicrobial treatments in both SPCs were prescribed by the designated farm veterinarian in compliance with Italian legislation (Legislative Decree 218/2023) and the European Union framework on veterinary medicinal products (Regulation (EU) 2019/6). After the weaning phase, pigs were transferred to growing–finishing farms (S3), where they were housed from approximately 30 kg to 160 kg of body weight, corresponding to the typical Italian production system for heavy pigs destined for dry-cured ham production. In both SPC1 and SPC2, animals were distributed into three pens per group with full slatted concreate floors, both farms had a natural ventilation system. Animals were fed ad libitum and fresh water was continuously available via nipple drinkers. Stocking density in all pens complied with the provisions of European Directive 2008/120/EC. Environmental enrichment remained consistent with the nursery phase and included the use of fixed iron chains combined with attached wooden blocks. Cleaning and disinfection protocols were consistent with those applied in the weaning units.

### Sampling and data collection

Biological samples were collected at five time points during the trial: at 21 days of age (T1), 10 days post-weaning (T2), one week before transfer to S3 (T3), 10 days after arrival in S3 (T4), and one week before slaughter (T5). At each time point, individual blood and faecal samples were collected from all pigs. Blood was drawn via jugular venepuncture into K3 EDTA tubes for haematological analysis, and into serum tubes containing a clot activator for serum separation. Serum tubes were left at room temperature for 2 h and centrifuged at 3000 × g for 10 min.

Faecal samples were collected directly from the rectum using sterile tubes, immediately frozen in liquid nitrogen, and stored at − 80 °C for subsequent microbiota and polyamine analyses. Faecal consistency was evaluated using a 5-point scale, with scores above 3.5 indicating diarrhoea. Additionally, at T2, T3, and T4, skin lesions were assessed in six body regions: ears, tail, neck, middle trunk, hindquarters, and flanks. Observations were conducted from a distance of 0.5 m within the pen, using a headlamp when necessary. Lesions were scored on a scale from 0 to 2 in accordance with the Welfare Quality^®^ (2009) protocol.

### Analysis of microbial profile

Faecal samples were used to extract the total bacterial DNA following the manufacturer’s instructions of the SPIN Kit for Soil (MP Biomedicals, Santa Ana, Ca, USA). DNA concentration and purity were controlled using a NanoDrop spectrophotometry (Fisher Scientific, Schwerte, Germany). DNA samples were then diluted and amplified for the V3-V4 region of the 16 S rRNA gene using the universal primers: TCGTCGGCAGCGTCAGATGTGTATAAGAGACAGCCTACGGGNGGCWGCAG and GTCTCGTGGGCTCGGAGATGTGTATAAGAGACAGGACTACHVGGGTATCTAATCC and the KAPA HiFi Hotstart Taq DNA Polymerase (KAPA Byosystems, Wilmington, MA, USA), then samples were sequenced using the Illumina NextSeq instruments. Libraries and sequencing were carried out using the MiSeq^®^ Reagent Kit V3-V4 on the MiSeq-Illumina^®^ platform. Microbial data analysis was carried out using the DADA2 pipeline [61], and taxonomy was assigned using the Silva Database (release 138.1) as a reference [62].

For metagenomic analysis, a total of 200 faecal samples were collected from a subset of 40 pigs (22 from SPC1 and 18 from SPC2), which were longitudinally sampled at five timepoints (T1 to T5). The same animals were followed and sampled throughout the study to assess temporal changes in the faecal microbiome. The subset was selected to ensure balance for litter of origin and initial body weight, allowing for consistent comparison between production chains over time. One faecal sample per pig was collected at each timepoint, resulting in a total of 200 samples (40 pigs × 5 timepoints). DNA samples were first fragmented to a size of 450–500 bp, then end-repaired and A-tailed using the FX enzyme mix according to the manufacturer’s thermal cycling instructions. The samples were incubated with DNA ligase and Illumina adapter barcodes at 20 °C for 15 min to enable adapter ligation. Following this, a purification step was conducted using Agencourt AMPure XP magnetic beads (Beckman Coulter, Brea, California, USA), and a 10-cycle PCR was performed for samples containing less than 100 ng of DNA. The DNA libraries were further purified, pooled to an equimolar concentration of 4 nM, and sequenced on an in-house Illumina NextSeq platform at the University of Bologna sequencing facility using a 2 × 150 bp paired-end protocol, generating over 3 Gb of data per sample.

### Analysis of faecal polyamines

The faecal concentrations biogenic amines (cadaverine, putrescine, spermidine and spermine; nmol/mL) were measured using high-performance liquid chromatography (HPLC) and quantified using fluorimetry as described by Correa et al. (2023) [12]. 

### Analysis of blood parameters

A total of 15 haematological parameters (erythrocyte traits: RBC, HGB, HCT, MCV, MCH, and MCHC); leukocyte traits: WBC, lymphocytes, neutrophils, eosinophils, basophils, and monocytes; platelet count (PLT) were detected using laser-empedimetric cytometry.

BAP, ROMs were assessed using the BAP and ROMs tests as described by Correa et al. 2023 [12]. Serum concentration of ceruloplasmin (ng/ml), serum amyloid (ng/ml), and haptoglobin (µg/ml), were assessed in the serum samples using a double-sandwich enzyme-linked iommunosorbent assay (ELISA) commercial kits (MyBioSource, San Diego, USA) according to the manufacturer’s instructions. Samples were diluted 1:1000 for serum amyloid and haptoglobin, and 1:100 for ceruloplasmin. Absorbance was set at 450 nm on the Multiskan multiplate reader (Mul- tiskanTM FC Microplate Photometer—Thermo Fisher Scientific) and concentrations were calculated using a four-point parametric curve.

Serum concentration of IgA, IgM and IgG was analysed using an Ig ELISA assay (Bethyl Laboratories, Montgomery, USA) following the protocol described by Luise et al. 2023 [63]. Prior to Immunoglobulins (Ig) analysis serum samples were incubated at 56 °C for 30 min. The reaction was quantified spectrophotometrically at an absorbance of 405 nm using a microplate reader (Multiskan FC Microplate Photometer – Thermo Fisher Scientific). The data regarding Igs concentrations were calculated using a four-point parametric curve and were expressed as milligram per millilitre (mg/mL).

### Statistical and bioinformatics analysis

#### Physiological parameters

The statistical analysis on blood parameters and polyamine concentration were carried out in R (v4.4.0), using the car (v3.1-2) lme4 (v1.1-35.3) and emmeans (v1.10.2) packages. The experimental unit for all analyses was the individual pig. Data on blood parameters, growth performance and polyamine concentration were analysed fitting the data using a linear mixed model including the type of SPCs (SPC1 or SPC2), the timepoint and their interaction as fixed factors and the litter of origin as random factor. Pairwise contrast was then performed within each timepoint using Tukey HSD. Data were then presented as estimated marginal means and standard error means. To examine the association between SPCs and the lesion score data, a Chi-square tests was conducted and the percentage of distribution of scores within each SPCs. Specifically, contingency tables were constructed for each combination of timepoint and body part, with rows representing groups and columns representing the observed scores (e.g., 0, 1, 2). Data on occurrence of diarrhoea were calculated assigning the diarrhoea status on pigs having a faecal score > 3, the statistical analysis was then conducted using a generalized mixed effect model, using a binomial distribution and including the type of SPCs (SPC1 or SPC2), the timepoint and their interaction as fixed factors and the litter of origin as random factor. Estimated means were then back transformed from the log scale to the response scale.

#### Microbial profile 16s RNA gene

Alpha (Shannon, Chao1 and InvSimpson indices) and Beta diversity (calculated as the Bray Curtis distance matrix), as well as the abundance of taxonomic categories, were analysed utilising R software 4.4.0 using the Phyloseq (v1.48.0) [64] and Vegan packages (v2.6-6.1) [65]. The Alpha diversity indices were analysed using an ANOVA model (*lmer* function) including the timepoint, the SPCs (SPC1 and SPC2), their interaction as factors and litter of origin as random effect. Pairwise contrasts were then performed within each timepoint using Tukey HSD. Beta diversity was analysed using a PERMANOVA Adonis test model (*adonis.test* function) which included the timepoint, the SPCs (SPC1 and SPC2) and their interaction in the model. Results were plotted using Principal coordinate analysis. Microbial markers within each timepoint were then identified using the LEfSe algorithm [66] at Genus level, only markers with a LDA score >3 and adjusted P value 0.05 were retained.

#### Shot-gun metagenomic

Species-level characterization of shotgun metagenomic data was conducted as it follows. Shotgun reads were first filtered using the standard operating procedures of the HMP Consortium [67], with the Sscrofa11.1 genome as reference for the host genome. The obtained reads were taxonomically characterized at species level by MetaPhlAn3 [68].

Metagenomes were functionally profiled using HUMAnN2 [69] to quantify abundance level of genes and pathways. Reads were aligned to sample-specific pangenomes, i.e., all gene families in any microorganism detected in a given sample, using Bowtie and the UniRef90, MinPath and KEGG databases [70–73]. Hits were counted per KEGG pathway and KO genes and normalized for length, alignment quality score and sequencing depth.

ARGs were identified from MAGs reconstructed from metagenomic reads. Open reading frames were predicted from MAG contigs using Prokka [74], dereplicated at 90% identity, and screened for antimicrobial resistance using the PathoFact pipeline [75]. High-confidence ARGs were retained based on “strict” and “perfect” matches determined by the Resistance Gene Identifier (RGI) against the Comprehensive Antibiotic Resistance Database (CARD) [76]. ARGs located on contigs classified as plasmid- or phage-derived were identified using PlasFlow and integrated phage detection tools and considered potentially mobile. ARGs were mapped to species-level genome bins (SGBs) using gene locus tags from Prokka annotations, and their abundance in each sample was estimated by combining their presence within SGBs with the relative abundance of the corresponding MAGs across samples. For detailed procedures on MAG and SGB reconstruction, we refer to Scicchitano et al. 2024 [55].

#### Core microbiota analysis

Core microbiota analysis was carried out by applying a prevalence and relative abundance of species data. Core taxa were defined as those taxa that reached a relative abundance of at least 1% in individual samples and were present in at least 50% of samples at each timepoint. A Venn diagram was then created to observe the shared core taxa determined by the intersection of these sets across timepoints.

#### Beta diversity and differential abundance analyses

Beta diversity was calculated, within each timepoint, at species, KO and ARG level using the Bray Curtis distance matrix and analysed using a PERMANOVA Adonis test model (*adonis.test* function) which included the SPCs (SPC1 and SPC2) as factor. Results were plotted using Principal coordinate analysis. Microbial markers within each timepoint were then identified using the LEfSe retaining only markers with a LDA score > 3 and P value 0.05. Regarding the functional profile a beta diversity matrix using Bray-Curtis distance matrix was calculated using KEGG pathway abundance data. KEGG pathway and ARGs gene associated with each SPCs, within each timepoint were then identified using DESEQ2, only pathways with log2FoldChange > 1.5 or < − 1.5 and P adjusted < 0.05 were retained.

#### Association between microbial profile and blood markers

To explore the relationship between host physiology and changes in gut microbiota composition over time, subjects were clustered based on various parameters of blood analysis, including basophils, eosinophils, neutrophils, WBC, lymphocytes, monocytes, PLT, MCV, Neutrophils to Lymphocytes ratio (NLR) and Lymphocytes to Monocyte ratio (LMR) to capture the synergistic interplay between innate and adaptive immunity [77]. Dynamic Time Warping (DTW) was utilized to compute the distance between each time series for each blood parameter in each animal. This was accomplished using the *dtw* R package v1.23-1 [78]. To handle missing data and associated varying time-series lengths, an Open-Begin-End DTW (OBE-DTW) approach was implemented, allowing for flexibility in the alignment of time series. Individuals with fewer than three time points available were discarded to ensure robust distance calculations.

A hierarchical clustering by using the *htclust* function in R cluster package v2.1.4 (https://CRAN.R-project.org/package=cluster) was subsequently applied to DTW distances in order to organize the data into clusters according to these temporal patterns. The optimal number of clusters for each parameter was determined using the silhoutte function in the same cluster package, which evaluates the quality of clustering by assessing the cohesion and separation of the clusters. Initially, clustering was performed separately for each physiological parameter to understand individual variable effects.

Each blood cluster was then included as a factor in the alpha diversity ANOVA model (using the *lm* function), which also considered the SPCs (SPC1 and SPC2) and timepoints as factors. The model additionally accounted for interactions between timepoints and blood clusters, and between the production chain and blood clusters. Pairwise contrasts were then performed within each timepoint using the Tukey HSD test. Beta diversity was analysed using a PERMANOVA Adonis test model (*adonis.test* function), which included the timepoint and the SPCs (SPC1 and SPC2) and the interactions between timepoint, blood cluster, and production chain. Finally, microbial markers associated for each blood cluster were then identified using the LEfSe algorithm at genus level; only markers with a LDA score > 4 and P value < 0.05 were retained.

## Supplementary Information

Below is the link to the electronic supplementary material.

Supplementary Material 1

## Data Availability

Sequencing data are available at European nucleotide archive under the project number PRJEB80533.

## Abbreviations

ADG: Average Daily Gain
AOPP: Advanced Oxidation Protein Products
ASV: Amplicon Sequence Variants
BAP: Biological Antioxidant Potential
BW: Body Weight
CER: Ceruloplasmin
DTW: Dynamic Time Warping
ELISA: Enzyme–Linked Immunosorbent Assay
HCT: Haematocrit
HGB: Haemoglobin
HP: Haptoglobin
IgA: Immunoglobulin A
IgG: Immunoglobulin G
IgM: Immunoglobulin M
KEGG: Kyoto Encyclopedia of Genes and Genomes
KO: KEGG Orthology
LDA: Linear Discriminant Analysis
LMR: Lymphocyte–to–Monocyte Ratio
MCH: Mean Corpuscular Haemoglobin
MCHC: Mean Corpuscular Haemoglobin Concentration
MCV: Mean Corpuscular Volume
NLR: Neutrophil–to–Lymphocyte Ratio
PCoA: Principal Coordinate Analysis
PWD: Post–Weaning Diarrheal
RBC: Red Blood Cell
ROMs: Reactive Oxygen Metabolites
SAA: Serum Amyloid A
SCFA: Short–Chain Fatty Acids
SEM: Standard Error of the Mean
SPC: Swine Production Chain
T1, T2, T3, T4, T5: Time Points 1 to 5
WBC: White Blood Cell
