# Supplementary material for: Early antimicrobial regimen shapes gut microbiota and health trajectories in pigs: a longitudinal study from weaning to finishing
Source: Anim Microbiome. 2025 Oct 24;7:110. doi: 10.1186/s42523-025-00477-x (PMC12553191; doi:10.1186/s42523-025-00477-x)
Supplement: Supplementary file 1 — Supplementary Material 1 [file 42523_2025_477_MOESM1_ESM.docx]

**Supplementary Table 1. Mean relative abundance and standard deviation of core microbiota taxa identified at each timepoint (T1–T5 ) in SPC1 and SPC2.** Core taxa were defined as species with a relative abundance ≥1% in at least 50% of samples within each timepoint. The table reports the mean relative abundance (%) and standard deviation (SD) calculated across all animals, without stratification by treatment group.

| **Time** | **Species** | **SPC1** | | **SPC2** | |
| --- | --- | --- | --- | --- | --- |
|  |  | **Abundance %** | **SD** | **Abundance %** | **SD** |
| T1 | s__Lactobacillus_vaginalis | 15.54 | 14.70 | 15.76 | 16.33 |
| T1 | s__Anaeromassilibacillus_sp_An172 | 9.77 | 9.37 | 15.25 | 13.86 |
| T1 | s__Methanobrevibacter_smithii | 7.49 | 6.00 | 8.07 | 5.81 |
| T1 | s__Lactobacillus_crispatus | 7.45 | 11.28 | 7.64 | 10.48 |
| T1 | s__Escherichia_coli | 6.60 | 11.45 | 3.52 | 4.43 |
| T1 | s__Lactobacillus_reuteri | 4.20 | 5.26 | 2.95 | 2.63 |
| T1 | s__Alistipes_shahii | 2.57 | 2.61 | 2.14 | 3.31 |
| T1 | s__Clostridium_clostridioforme | 1.99 | 2.45 | 1.83 | 1.28 |
| T1 | s__Butyricimonas_virosa | 1.92 | 1.36 | 1.23 | 0.93 |
| T1 | s__Ruminococcaceae_bacterium_D5 | 1.29 | 1.10 | 2.61 | 3.01 |
| T1 | s__Phascolarctobacterium_succinatutens | 1.19 | 1.62 | 3.24 | 3.11 |
| T2 | s__Lactobacillus_amylovorus | 50.33 | 20.72 | 53.62 | 19.95 |
| T2 | s__Lactobacillus_reuteri | 7.43 | 5.49 | 11.57 | 6.43 |
| T2 | s__Megasphaera_elsdenii | 7.38 | 8.94 | 1.68 | 2.43 |
| T2 | s__Prevotella_copri | 3.59 | 5.72 | 2.98 | 2.78 |
| T3 | s__Lactobacillus_amylovorus | 52.88 | 13.57 | 37.28 | 11.66 |
| T3 | s__Lactobacillus_reuteri | 9.30 | 3.57 | 14.81 | 4.69 |
| T3 | s__Butyricicoccus_porcorum | 6.85 | 6.81 | 0.70 | 1.58 |
| T3 | s__Prevotella_copri | 6.62 | 4.77 | 7.65 | 4.64 |
| T3 | s__Megasphaera_elsdenii | 5.69 | 4.54 | 10.50 | 5.28 |
| T3 | s__Lactobacillus_johnsonii | 2.10 | 2.74 | 10.63 | 5.36 |
| T3 | s__Coprococcus_catus | 1.51 | 1.01 | 1.17 | 1.04 |
| T3 | s__Phascolarctobacterium_succinatutens | 1.48 | 0.91 | 1.30 | 0.66 |
| T4 | s__Lactobacillus_amylovorus | 37.46 | 13.51 | 38.79 | 12.02 |
| T4 | s__Lactobacillus_reuteri | 14.98 | 5.32 | 11.67 | 6.02 |
| T4 | s__Butyricicoccus_porcorum | 9.68 | 10.11 | 2.70 | 3.14 |
| T4 | s__Prevotella_copri | 6.30 | 4.82 | 5.30 | 4.83 |
| T4 | s__Megasphaera_elsdenii | 5.76 | 3.15 | 11.28 | 4.21 |
| T4 | s__Lactobacillus_johnsonii | 5.63 | 3.84 | 0.92 | 1.13 |
| T4 | s__Phascolarctobacterium_succinatutens | 1.64 | 1.15 | 3.74 | 1.88 |
| T4 | s__Prevotella_sp_CAG_520 | 1.45 | 0.59 | 0.72 | 0.45 |
| T4 | s__Collinsella_aerofaciens | 0.80 | 0.74 | 2.70 | 2.14 |
| T5 | s__Lactobacillus_amylovorus | 41.02 | 24.63 | 42.20 | 17.85 |
| T5 | s__Turicibacter_sanguinis | 11.50 | 13.99 | 4.11 | 5.24 |
| T5 | s__Lactobacillus_reuteri | 10.57 | 4.70 | 6.56 | 4.71 |
| T5 | s__Methanobrevibacter_smithii | 8.68 | 8.02 | 11.06 | 5.42 |
| T5 | s__Treponema_porcinum | 6.37 | 6.26 | 4.24 | 3.60 |
| T5 | s__Prevotella_sp_P5_92 | 3.97 | 3.44 | 1.44 | 1.09 |
| T5 | s__Coprococcus_catus | 1.17 | 1.63 | 1.58 | 1.21 |
| T5 | s__Prevotella_copri | 1.14 | 1.07 | 4.28 | 6.13 |

**Supplementary Table 1.** Composition of the experimental diets during the weaning and growing finishing period

| Ingridients, % | **Weaning phase** | | | **Growing-Finishing phase** | | | |
| --- | --- | --- | --- | --- | --- | --- | --- |
|  | **6-10 kg BW** | **10-20 kg BW** | **20-30 kg BW** | **30-50 kg BW** | **50-90 kg BW** | **90-130 kg BW** | **130-170 kg BW** |
| Corn | 20.32 | 24.92 | 29.01 | 42.78 | 45.39 | 46.61 | 46.09 |
| Wheat | 6.00 | 20.00 | 25.00 | 11.00 | 11.00 | 15.00 | 16.00 |
| Barley | 16.00 | 15.00 | 12.00 | 4.00 | 5.00 | 6.00 | 12.00 |
| Cooked Cereals | 15.00 | - | - | - | - | - | - |
| Sweet Whey Powder | 7.00 | 6.00 | - | - | - | - | - |
| Soybean Meal | 6.00 | 9.00 | 13.00 | 11.50 | 8.00 | 9.00 | 3.50 |
| Soybean Protein Concentrate | 6.00 | 4.00 | - | - | - | - | - |
| Wheat Bran | 5.00 | 5.00 | 6.00 | 7.00 | 8.00 | 9.50 | 10.00 |
| Full Fat Soybean | 5.00 | 6.00 | 6.00 | - | - | - | - |
| Whey Protein Concentrate | 3.00 | - | - | - | - | - | - |
| Lignocellulose | 2.00 | 1.50 | 0.8 | - | - | - | - |
| Soybean Oil | 2.00 | 2.00 | 3 | - | - | - | - |
| Coconut Oil | 1.6 | 1.6 | - | - | - | - | - |
| Limestone | 0.95 | 1 | 0.96 | 1.25 | 1.20 | 1.20 | 1.20 |
| Premix Vitamin/Mineral^1^ | 0.50 | 0.50 | 0.35 | 0.25 | 0.25 | 0.20 | 0.18 |
| Benzoic Acid | 0.50 | 0.50 | - | 0.50 | - | - | - |
| Sodium Chloride | 0.30 | 0.30 | 0.4 | 0.40 | 0.40 | 0.40 | 0.40 |
| Sodium Butyrate | 0.30 | 0.20 | 0.1 | - | - | - | - |
| Sodium Bicarbonate | 0.15 | 0.15 | 0.15 | 0.00 | 0.15 | 0.15 | 0.15 |
| Flavour and Sweetener | 0.10 | 0.08 | 0.05 | - | - | - | - |
| Probiotic | 0.10 | 0.08 | 0.06 | - | - | - | - |
| Choline Chloride (75%) | 0.08 | 0.07 | 0.07 | 0.04 | 0.05 | 0.03 | - |
| Calcium Phosphate | - | - | 0.45 | 0.30 | 0.25 | 0.25 | 0.10 |
| **Total** | 100.00 | 100.00 | 100.00 | 100.00 | 100.00 | 100.00 | 100.00 |

^1^Premix provided the following per kilogram of premix: Vitamin A 2,600,000 UI, Vitamin D₃ 800,000 UI, Vitamin E 10,000 mg, Vitamin B₁ 600 mg, Vitamin B₂ 1,600 mg, Vitamin B₆ 1,200 mg, Vitamin B₁₂ 8 mg, Vitamin PP 14,000 mg, Vitamin K₃ 840 mg, Folic Acid 860 mg, Biotin 120 mg, Pantothenic Acid 8,400 mg, Zn 28,000 mg, Mn 19,200 mg, Cu 5,000 mg, Fe 36,200 mg, I 690 mg, Se 120 mg, Xylanase 1,440,000 EPU, 6-Phytase 300,000 FTU.

**Supplementary Table 2.** Nutritional composition of the experimental diets during the weaning and growing finishing period

| **Component** | **Unit** | **Weaning phase** | | | **Growing-Finishing phase** | | | |
| --- | --- | --- | --- | --- | --- | --- | --- | --- |
|  |  | **6-10 kg BW** | **10-20 kg BW** | **20-30 kg BW** | **30-50 kg** | **50-90 kg** | **90-130 kg** | **130-170 kg** |
| Dry Matter | % | 89.82 | 89.55 | 88.46 | 87.80 | 87.64 | 87.39 | 87.80 |
| Moisture | % | 10.18 | 10.45 | 11.54 | 12.20 | 12.36 | 12.61 | 12.20 |
| Net Energy | MJ | 10.07 | 10.06 | 10.01 | 9.65 | 9.68 | 9.44 | 9.38 |
| Metabolizable Energy | MJ | 13.53 | 13.59 | 13.52 | 13.18 | 13.21 | 12.99 | 12.93 |
| Crude Protein | % | 17.60 | 17.59 | 17.20 | 16.12 | 14.77 | 13.98 | 12.21 |
| Crude Fat | % | 6.37 | 6.46 | 6.01 | 4.84 | 4.61 | 3.36 | 3.01 |
| Crude Fiber | % | 4.45 | 4.17 | 3.88 | 3.50 | 3.49 | 3.96 | 4.22 |
| Starch | % | 34.64 | 35.54 | 39.55 | 41.09 | 43.50 | 45.08 | 48.41 |
| Sugars | % | 8.48 | 7.26 | 3.19 | 3.83 | 3.69 | 3.81 | 2.59 |
| Phosphorus | % | 0.40 | 0.40 | 0.46 | 0.49 | 0.46 | 0.48 | 0.44 |
| Calcium | % | 0.67 | 0.68 | 0.73 | 0.65 | 0.60 | 0.62 | 0.57 |
| Sodium | % | 0.26 | 0.26 | 0.23 | 0.23 | 0.25 | 0.25 | 0.23 |
| Potassium | % | 0.84 | 0.83 | 0.74 | 0.79 | 0.74 | 0.73 | 0.59 |
| Magnesium | % | 0.19 | 0.19 | 0.19 | 0.20 | 0.19 | 0.19 | 0.18 |
| Chlorides | % | 0.80 | 0.65 | 0.53 | 0.47 | 0.43 | 0.44 | 0.37 |
| Crude Ash | % | 5.44 | 5.18 | 4.96 | 4.59 | 4.41 | 4.50 | 4.05 |
| Lysine | % | 1.57 | 1.53 | 1.42 | 1.14 | 0.96 | 0.84 | 0.70 |
| Methionine | % | 0.62 | 0.60 | 0.54 | 0.42 | 0.33 | 0.26 | 0.20 |
| Threonine | % | 0.96 | 0.93 | 0.87 | 0.71 | 0.57 | 0.53 | 0.45 |
| Tryptophan | % | 0.31 | 0.30 | 0.28 | 0.21 | 0.17 | 0.16 | 0.14 |
| Valine | % | 1.06 | 1.05 | 0.98 | 0.72 | 0.64 | 0.63 | 0.55 |
| SID Lysine | % | 1.46 | 1.42 | 1.31 | 1.02 | 0.85 | 0.73 | 0.60 |
| SID Methionine | % | 0.59 | 0.57 | 0.51 | 0.39 | 0.30 | 0.23 | 0.17 |
| SID Threonine | % | 0.86 | 0.84 | 0.78 | 0.61 | 0.47 | 0.44 | 0.36 |
| SID Tryptophan | % | 0.28 | 0.27 | 0.25 | 0.18 | 0.14 | 0.13 | 0.11 |
| SID Valine | % | 0.95 | 0.94 | 0.87 | 0.61 | 0.54 | 0.52 | 0.45 |
| Vitamin A | IU | 16000.00 | 16000.00 | 11200.00 | 6500.00 | 6500.00 | 5200.00 | 4680.00 |
| Vitamin D3 | IU | 2000.00 | 2000.00 | 1400.00 | 2000.00 | 2000.00 | 1600.00 | 1440.00 |
| Vitamin E | mg | 80.00 | 80.00 | 56.00 | 25.00 | 25.00 | 20.00 | 18.00 |
| Vitamin B1 | mg | 3.00 | 3.00 | 2.10 | 1.50 | 1.50 | 1.20 | 1.08 |
| Vitamin B2 | mg | 6.00 | 6.00 | 4.20 | 4.00 | 4.00 | 3.20 | 2.88 |
| Vitamin B6 | mg | 4.00 | 4.00 | 2.80 | 3.00 | 3.00 | 2.40 | 2.16 |
| Vitamin B12 | mg | 0.05 | 0.05 | 0.04 | 0.02 | 0.02 | 0.02 | 0.01 |
| Vitamin B3 | mg | 80.00 | 80.00 | 56.00 | 35.00 | 35.00 | 28.00 | 25.20 |
| Vitamin B5 | mg | 21.00 | 21.00 | 14.70 | 21.00 | 21.00 | 16.80 | 15.12 |
| Biotin | mg | 0.30 | 0.30 | 0.21 | 0.30 | 0.30 | 0.24 | 0.22 |
| Folic Acid | mg | 3.84 | 3.84 | 2.69 | 2.16 | 2.16 | 1.73 | 1.56 |
| Vitamin K3 | mg | 2.94 | 2.94 | 2.06 | 2.10 | 2.10 | 1.68 | 1.51 |


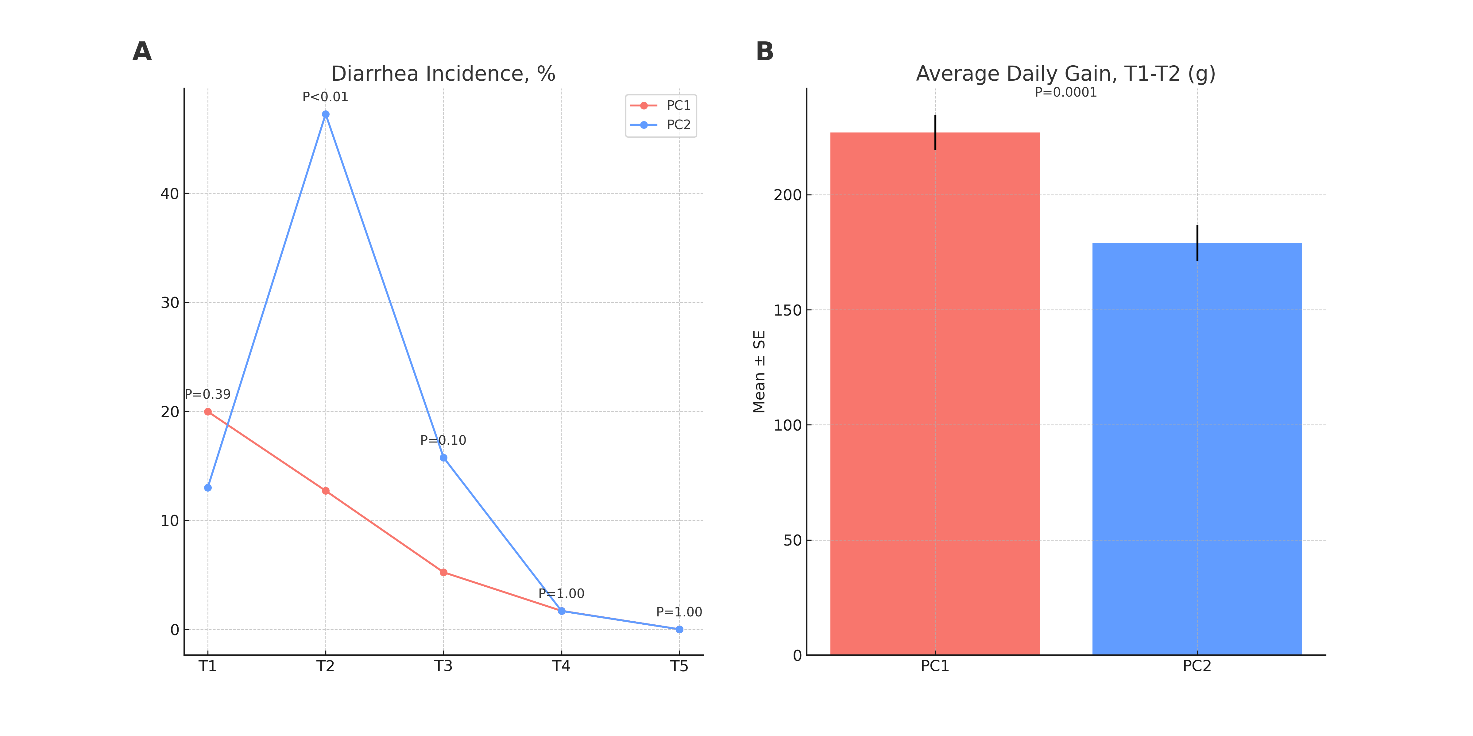


**Supplementary Figure 1.** Line plot displaying the proportion of pigs showing diarrhea at timepoints T2 to T5 in SPC1 and SPC2. Diarrhea was assessed based on fecal scores >3.5 (**A**). Average daily gain (ADG) from T1 to T2 in pigs from both production chains (**B**).


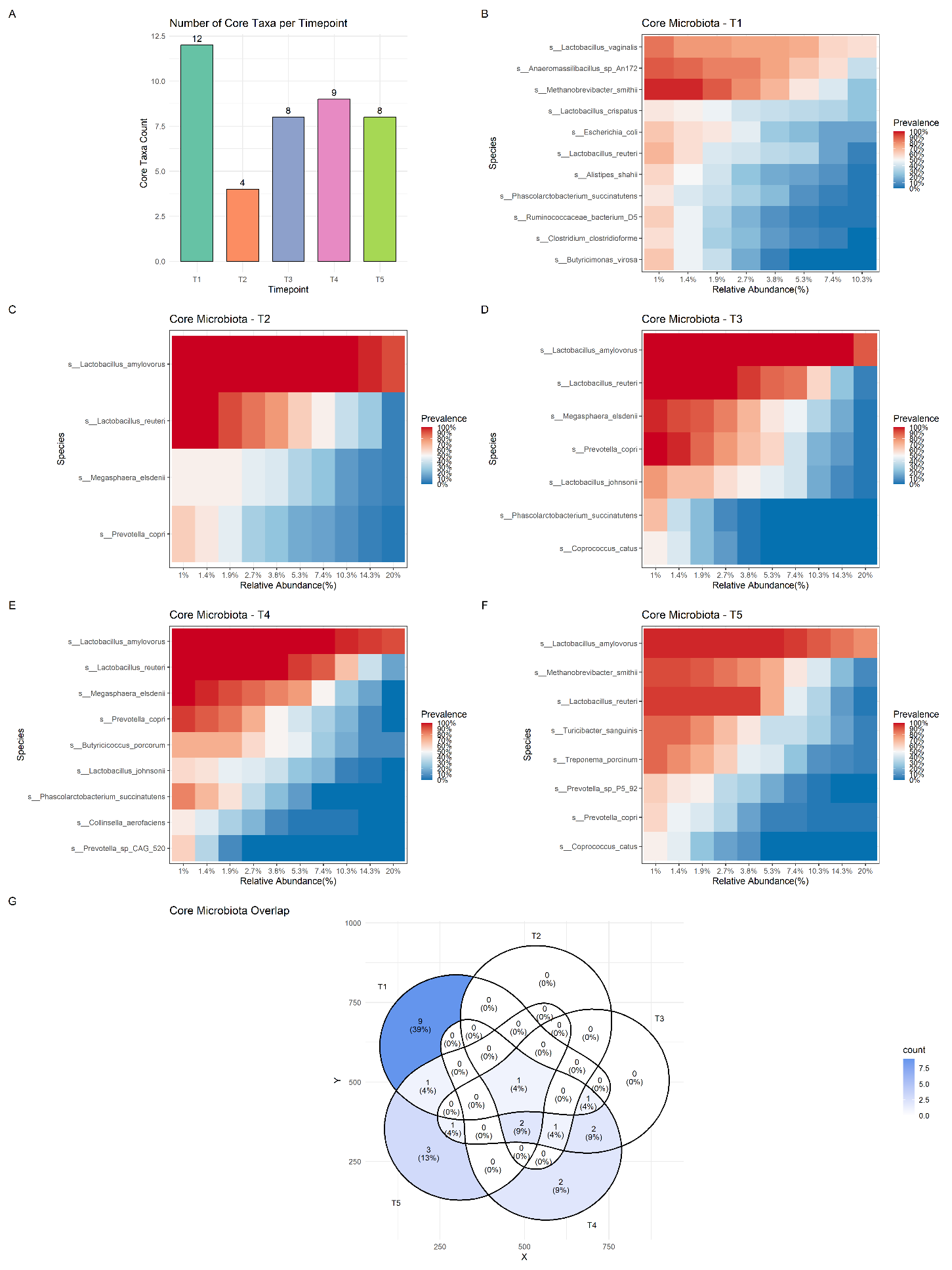


**Supplementary Figure 2.** Overview of Core Microbiota Dynamics Over Time. Panel **A** shows a bar plot quantifying the number of core taxa present at each timepoint. Panels **B** through **F** present heatmaps for timepoints T1, T2, T3, T4, and T5, respectively, illustrating the relative abundances of microbial genera across a range of detection thresholds. Panel **G,** is a Venn diagram depicting the overlap of core taxa among the different timepoints.
